# Chiral Ligand-Protected Gold Nanoclusters as Biosensors for Small Chiral Biomolecules: A Computational Study

**DOI:** 10.1021/acsnano.5c20222

**Published:** 2026-02-25

**Authors:** Zohreh Fallah, Sami Malola, María Francisca Matus, Hannu Häkkinen

**Affiliations:** † Department of Physics, Nanoscience Center, 4168 University of Jyväskylä, Jyväskylä FI-40014, Finland; ‡ Department of Chemistry, Nanoscience Center, University of Jyväskylä, Jyväskylä FI-40014, Finland

**Keywords:** chirality, gold nanoclusters, circular dichroism, enantiospecific binding, molecular dynamics

## Abstract

Detection of chiral biomolecules in biological environments presents an important challenge: to develop sensitive, noninvasive sensors that Could have an impact in several areas such as drug discovery, diagnostics of diseases, and care. In this work, we introduce a strategy for experimentally realizable, noninvasive sensing of small chiral biomolecules in aqueous solvents, validated via classical force-field molecular dynamics simulations and density functional theory calculations. We investigated the interactions of the L/D forms of glutathione and seven chiral amino acids (Ala, Arg, Asp, Cys, Glu, Ser, Tyr) with six chiral, water-soluble, thiolate-protected gold nanoclusters in the range of 25–144 gold atoms, via dynamical sampling extending up to 3 μs time scales in water at neutral pH. We found surprisingly large variations in the binding probability (from <1% to 100%) of these analytes to the nanoclusters, with the dominating interaction being electrostatics between the analyte and the nanoclusters’ ligand surface, augmented by hydrogen bonding and van der Waals interactions. Computed circular dichroism spectra for several nanocluster–analyte complexes predict the identification of analyte-specific adsorption events and even the resolution of the adsorbed enantiomer in selected cases, constituting an experimentally realizable sensing function. Our results suggest that chiral ligand-protected gold nanoclusters could be used for noninvasive chiral sensing, creating a tunable toolbox where the nanocluster size and chiral ligand type could be varied for optimizing the sensing activity for specific targets.

## Graphical abstract

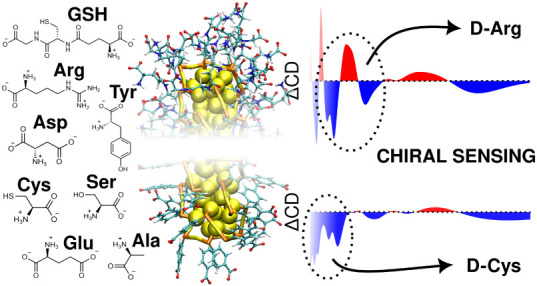

## 1 Introduction

Chirality is a fundamental feature of life.^1–3^ Most biomolecules are chiral and have a preferred handedness, such as the l-form of amino acids forming proteins and the d-form of sugars found in DNA and RNA. The origin of this molecular asymmetry and its role in the emergence of life remain among the most intriguing questions in chemistry and biology. Therefore, understanding how a chiral molecule of a specific handedness functions in a biological environment is of central importance. Even minor deviations from homochirality can have significant consequences for biological processes. Small amounts of biomolecules with the opposite configuration, known as “minority enantiomers”, are often associated with physiological disorders or diseases. For instance, d-amino acids, either free or within peptides and proteins, have been linked to aging-related processes, cancer, various neurological or renal pathologies, and the clinical progression of infectious diseases.^4–9^ These findings highlight d-amino acids as promising biomarkers for diagnostics and as targets for drug discovery.^10–15^

The importance of chirality extends well beyond that of natural biomolecules. In the pharmaceutical industry, more than 90% of drugs are chiral or mixtures of chiral compounds, while only one enantiomer has the desirable biological activity.^1^ Therefore, the ability to distinguish and quantify specific enantiomers in complex biological environments is essential for both fundamental research and applied medicine. Developing sensitive and noninvasive sensors that can work reliably in biological environments and detect individual enantiomers would not only deepen our understanding of biological chirality but also open pathways for disease diagnosis, treatment, and precision medicine.

Small noble metal nanoclusters with up to a 2 nm metal core size and a stabilizing layer of organic molecules at the surface form an interesting class of nanomaterials that have potential for sensing applications. Their metallic core (gold, silver, copper, or their mixtures) has quantized electronic states, which lead to discrete photophysical properties in emission and absorption.^16–24^ The solubility and interactions between the nanoclusters and biological targets in aqueous environments can be tuned via the chemical properties of the protecting molecular layer. In recent years, several experimental and computational studies have addressed nanocluster–biomolecule hybrids, investigating their binding and biomolecules’ impact on the electronic structure of the nanocluster and the ensuing photophysics of absorption and emission that could be used to constitute a sensing function.^25–33^ Other studies have investigated the potential of these nanoclusters to carry photosensitizers that would signal changes in the local chemical environment in “working” biological environments, such as live cells.^34,35^

A great advantage of using noble metal nanoclusters as prototypes for sensing chiral objects is that experimental structural information exists for hundreds of nanoclusters, particularly when the metal core is made of gold. Many such nanoclusters either have an intrinsic chiral structure, even with an achiral ligand, or can be made chiral when the metal core is stabilized with a chiral ligand. Hybrids between chiral nanoclusters and chiral targets are, therefore, ideal systems to investigate by using atomistic simulation tools. Here, we leverage the hypothesis that chirality at the nanocluster’s surface may give rise to enantiospecific adsorption of chiral objects that would change the nanocluster’s circular dichroism (CD) absorption spectrum. We studied the interaction between six gold nanoclusters (AuNCs), ranging from 25 to 144 atoms, and eight small chiral biomolecules. The nanoclusters are stabilized with two different water-soluble ligands: chiral l-glutathione (l-GSH) and achiral *para*-mercaptobenzoic acid (*p*MBA). The specific compositions studied were: Au_25_(GSH)_18_, Au_38_(GSH)_24_, Au_102_(GSH)_44_, Au_38_(*p*MBA)_24_, Au_102_(*p*MBA)_44_, and Au_144_(*p*MBA)_60_. The structures of all these AuNCs are based either on a known crystal structure with the specific ligand or on a crystal structure with another thiolate. As analytes, we studied the l- and d- forms of GSH and seven chiral amino acids: alanine (Ala), arginine (Arg), aspartate (Asp), cysteine (Cys), glutamate (Glu), serine (Ser), and tyrosine (Tyr). Our guiding questions were: (i) Are there variations in the binding strength, reflected in the binding probability? In other words, does one observe chemical selectivity for targets? (ii) Are there variations in enantiospecific binding? (iii) Are there noticeable changes in the nanoclusters’ CD response induced by the binding of the analyte to detect binding? (iv) Is one able to distinguish the analyte’s handedness by examining the CD spectrum? We addressed questions (i) and (ii) by extensive sampling of nanocluster–analyte interactions by molecular dynamics (MD) simulations in water for 96 nanocluster–analyte systems, performing over 300 MD simulations for up to 1.5 μs time scale for each complex. Questions (iii) and (iv) were addressed via density functional theory (DFT) calculations of the CD response for a selection of nanocluster–analyte hybrids. We indeed found surprisingly large variations in the binding probability (from close to 0–100%) for the eight analytes binding to the nanoclusters and analyzed the underlying interactions. DFT calculations indicate that, for selected cases, even the handedness of the chiral analyte can be detected from the CD spectrum of the nanocluster–analyte hybrid. Our work thus predicts that the concept of using chiral gold nanoclusters as chiral sensors in aqueous solutions is viable and worth experimental efforts for realization.

## 2 Results and Discussion

### 2.1 Studied Systems and Their Chemical Selectivity for Analyte Binding

Figure 1 shows the atomic visualization of the six chiral AuNCs with their chemical compositions and the eight chiral analytes in their l-form. Our data set includes 96 systems, each comprised of one nanocluster and one analyte (6 nanoclusters × 8 analytes × 2 analyte enantiomers). For each system, three independent MD simulations spanning 500 ns were performed under standard conditions in a water box (technical details of the simulations and data analysis are discussed in the Methods section). In addition, concentration effects of analytes were studied by extending the MD simulations to cover 12 additional systems. These included the analytes l -Arg, d-Arg, l-Tyr, and d-Tyr with the Au_38_(*p*MBA)_24_ nanocluster, as well as l-Arg and d-Arg with the Au_38_(GSH)_24_ nanocluster, in which either 10 or 20 analytes were included in the simulation box. These extended simulations were run up to 1 μs each. Hence, the total number of MD simulations conducted was 324. In the following discussion, we adopt a shorthand notation for nanocluster–analyte complexes; for example, l-Arg interacting with the Au_38_(*p*MBA)_24_ nanocluster is referred to as 38*p*MBA/l-Arg, thereby avoiding the repetition of nonessential information.

**Figure 1:**
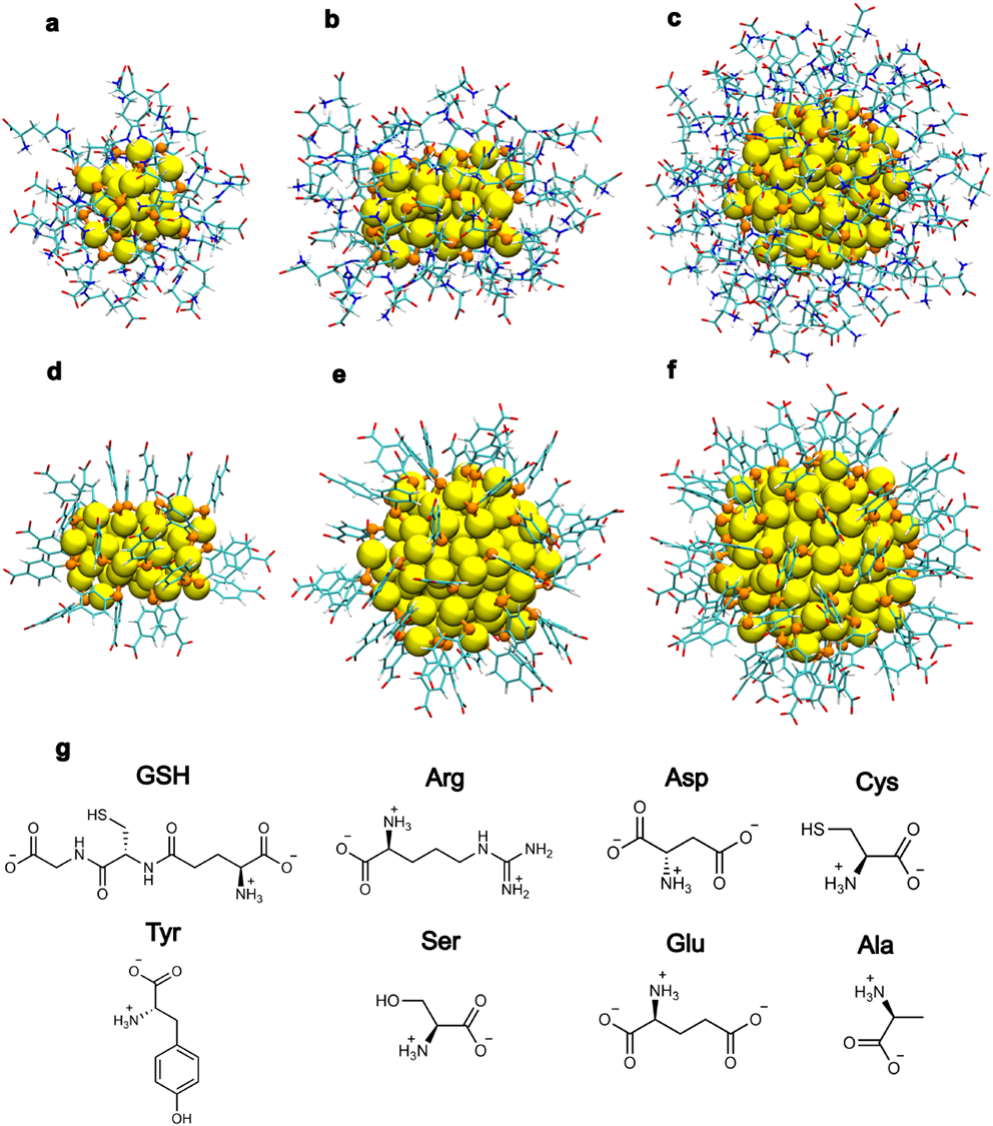
Visualization of the studied systems. Atomic structures of (a) Au_25_(GSH)_18_, (b) Au_38_(GSH)_24_, (c) Au_102_(GSH)_44_, (d) Au_38_(pMBA)_24_, (e) Au_102_(pMBA)_44_ and (f) Au_144_(pMBA)_60_. Color code: Au = yellow; S = orange; O = red; N = blue; H = white; C = cyan. Panel (g) presents the L forms of the analytes in their zwitterionic state at pH 7.

The adsorption probability parameter (*P*, in %) indicates the likelihood of analyte adsorption in either l- or d-forms on gold nanoclusters (AuNCs). *P* values greater than 20% are highlighted using a color scale ranging from dark green (highest P) to faint green. The data are compiled from molecular dynamics simulations with one analyte per system.

Table 1 shows the summary of chemical selectivity for analyte binding in terms of the binding probability, *P*, which is defined as the percentage of the full simulation time (including all replicas) during which a given analyte and the nanocluster had at least one chemical contact, as described in the Supporting Information. In Table 1, only one analyte per simulation is considered.

**Table 1:**
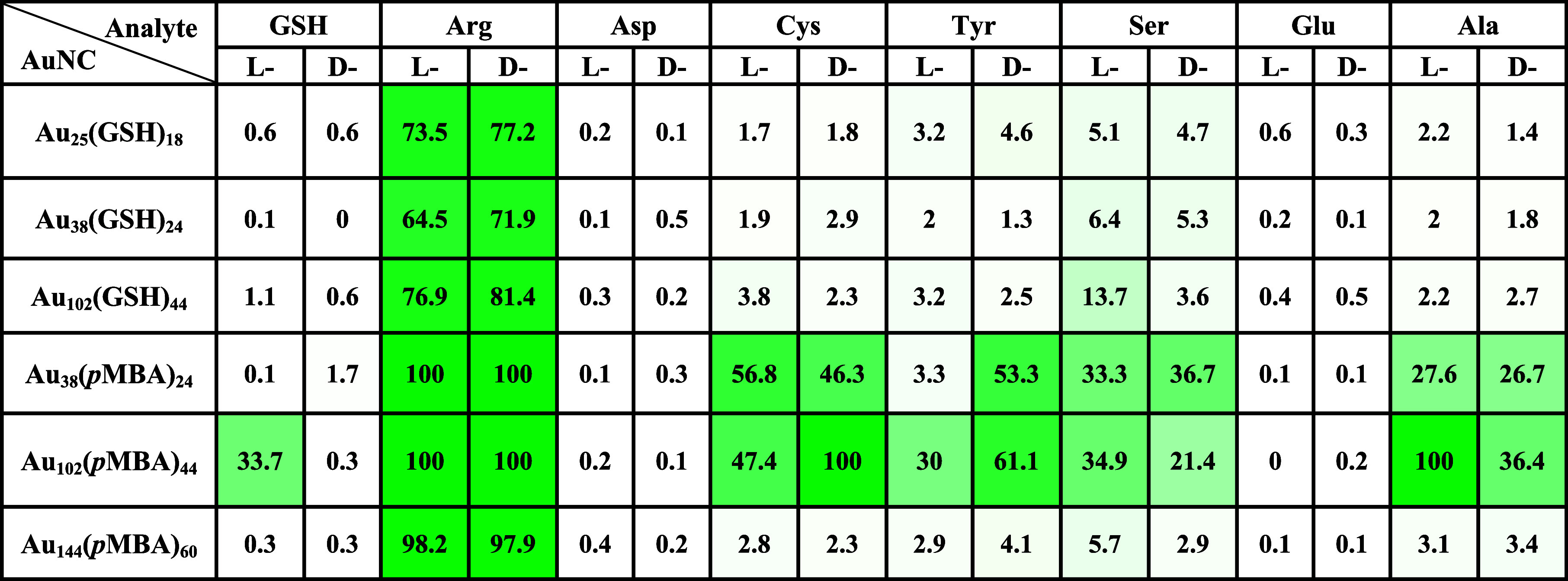
Binding Probability of l/d-Analytes on Chiral Gold Nanoclusters

### 2.2 GSH-Protected Nanoclusters and Arginine

Considering nanoclusters that are protected by the l-GSH ligand, only arginine has consistently high *P* values in the range of 64.5% < *P* < 81.4% in both l- and d-forms, with 64.5% corresponding to 38GSH/l-Arg and 81.4% corresponding to 102GSH/d-Arg. The data also reveal that adsorption of d-Arg is slightly favored over l-Arg in all three GSH-protected nanoclusters. All the other analytes have *P* values <14% and are not discussed here in detail.

The center-of-mass (COM) distances between l/d-Arg and GSH-protected nanoclusters are shown in Figure 2 for all systems with one analyte. In all runs, we observed variations of the COM in the range of ∼1–∼10 nm, indicating that analyte binding and desorption are continuous dynamical effects. The average lowest values of COM are 0.99 nm for 25GSH, 1.07 nm for 38GSH, and 1.41 nm for 102GSH, implying binding of the analyte and are in line with the increasing size of the gold nanocluster. We also evaluated other indicators for nanocluster–analyte interactions, namely the number of hydrogen bonds (HB), the Coulomb energy, and the van der Waals interaction described by the Lennard–Jones (LJ) part of the total interaction energy. This data is shown in Figure S1 (Supporting Information). We observed that the HB number fluctuates in a large range between 0 and 12 in all runs, and Coulomb energy has a dominant role over the LJ interaction. This can be understood because, under the simulation conditions corresponding to pH 7, both the GSH ligand shell and arginine are charged.

**Figure 2:**
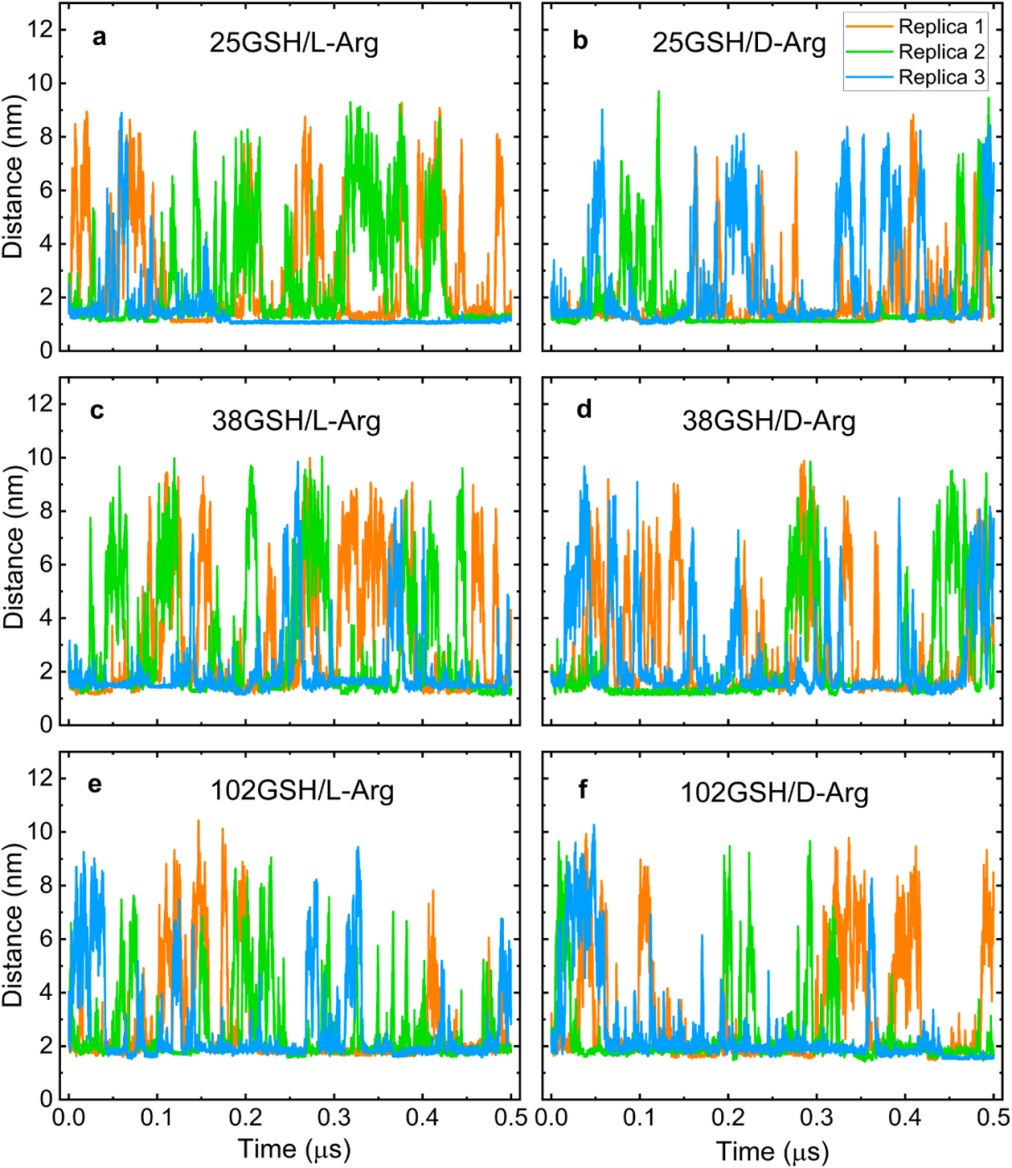
Time evolution of the center-of-mass distance between l/d-Arg and GSH-protected nanoclusters. Data from three independent 0.5 μs molecular dynamics simulations are shown. ( a, b) 25-atom gold clusters, (c, d) 38-atom gold clusters, (e, f) 102-atom gold clusters.

Figure 3 shows selected structures from the MD trajectories for l/d-Arg with 25GSH, 38GSH, and 102GSH. We observed that l/d-Arg binds, in most cases, to the surface of the GSH ligand shell. The interaction of arginine with the surface is mainly through its side chain—the positively charged guanidine group (HNC­(NH_2_)_2_^+^), and its amino group (−NH_3_^+^) with the carboxyl (COO^–^) and carboxamide group (−C­(=O)-NH−) of the different GSH peptides. There is also interaction between the carboxyl group of arginine and the amino and carboxamide groups of the different GSH ligands. In one case (Figure 3b) we observed a very different binding mode where the amino group penetrates deeply between the GSH ligand shell and gets close to the gold core.

**Figure 3:**
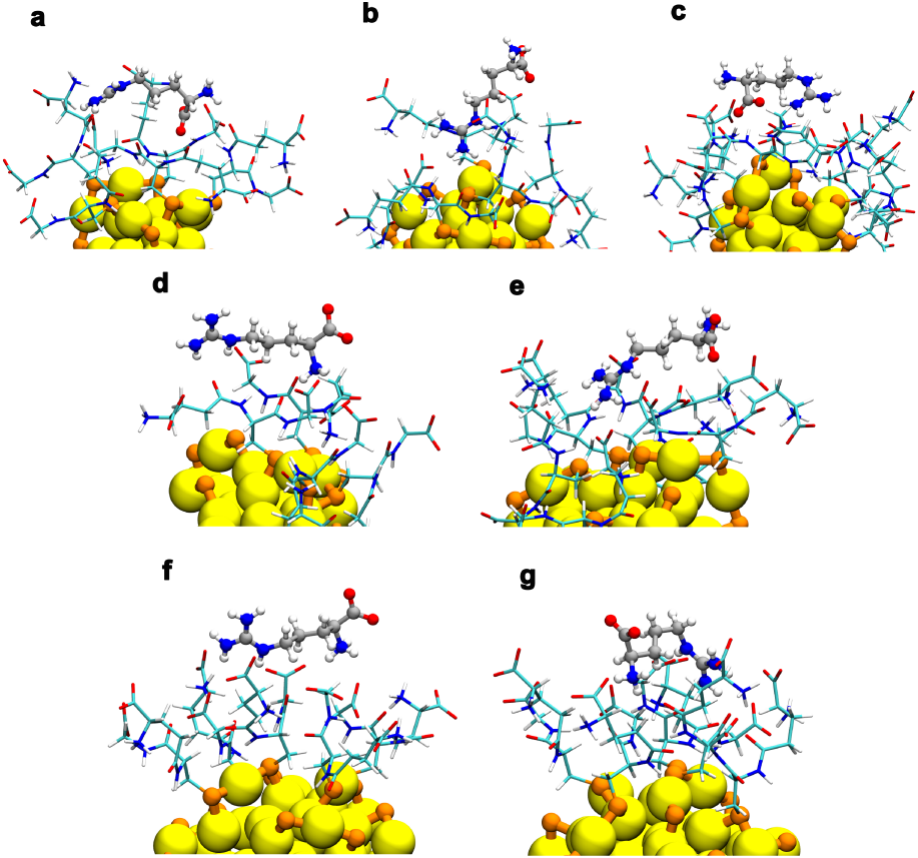
Representative structures of l/d-Arg interacting with GSH-protected nanoclusters. The structures were obtained from a 1.5 Å RMSD-based geometric clustering analysis (see Methods section) applied to trajectories from three independent molecular dynamics replicas. (a, b) l-Arg and (c) d-Arg with Au_25_(GSH)_18_, (d) l-Arg and (e) d-Arg with Au_38_(GSH)_24_, (f) l- Arg and (g) d-Arg with Au_102_(GSH4)_44._ Color code: Au = yellow; S = orange; O = red; N = blue; H = white; C (ligand layer)=cyan; C (analyte)=gray.

In addition to single-analyte MD simulations, we investigated the concentration effects for binding of l/d-Arg to the 38GSH nanocluster with extended time-scale (1 μs) MD simulations, with either 10 or 20 analytes in the simulation box. Figure S2a–d shows the time evolution of the number of bound l/d-Arg in both the 10-analyte and 20-analyte systems. In the 10-analyte systems, the average number of bound l/d analytes is about 6.4/6.6 (i.e., a binding probability *P* ≈ 65%), while it is around 11.2/11.4 (*P* ≈ 56%) for the 20-analyte systems. l- and d- forms of arginine bind with very similar probabilities. Arginine molecules interact mostly via their guanidine and amino groups with the ligand layer. As visualized by selected snapshots of MD simulations in Figures S2e–h, both l- and d-Arg molecules also interact with each other via their amino, guanidine, and carboxyl groups. This seems to enhance their binding to the nanocluster. Aggregation of arginine can be observed on the nanocluster surface, especially in the systems with 20 l-Arg and 20 d-Arg, as shown in Figure S2i,j. Similar to the case of single l/d-Arg discussed above, HBs play an important role in binding to the nanocluster, and the Coulomb interaction energy dominates over the LJ energy (Figure S3).

### 2.3 pMBA-Protected Nanoclusters with Analytes

Table 1 shows that *p*MBA-protected nanoclusters exhibit larger variations in the chemical selectivity of binding to various analytes compared with GSH-protected nanoclusters. Hence, we discuss the interactions of one cluster type at a time.

The 38*p*MBA nanocluster displays moderate to very strong interactions for several analytes in terms of the binding probability: about 27% for l/d-Ala, 33.3% and 36.7% for l-Ser and d-Ser, respectively, 53.3% for d-Tyr, 56.8% and 46.3% for l-Cys and d-Cys, respectively, and 100% for L/d-Arg. The dynamical variations of COM distances between the analytes and the nanocluster are shown in Figure S4a–j, HB data in Figure S5, and Coulomb and LJ energies in Figure S6. Representative bound configurations of the above-mentioned analytes to 38*p*MBA are shown in Figure S7. A full discussion on the details of the observed interactions between the analytes and the nanocluster is found in the Supporting Information. Here, we noted that the *p*MBA-protected nanocluster allows, in most cases, the analytes to penetrate the ligand layer and have direct contact with the gold–thiolate interface, as opposed to the GSH-protected nanoclusters discussed above. We also note that there is a surprisingly clear enantioselectivity in the binding of l-Tyr (*P* = 3.3%) and d-Tyr (*P* = 53.3%). Concentration effects were studied in the case of l/d-Arg and l/d-Tyr (Figure S8). We observed that l/d-Arg binding is almost 100%,% even in the case of runs containing 10 analytes in the simulation box (9.8/9.7, i.e., 97% analytes bound to the nanocluster), and 20-analyte runs increase the average number of bound l/d-Arg to about 14.8/14.9 (*P* = 74%) molecules per nanocluster. By analyzing the corresponding MD data for l/d-Tyr, we observed that the enantioselectivity seems to be lost when the number of analytes in the simulation box is increased, and doubling the analyte concentration from 10 to 20 molecules per nanocluster does not increase the average number of bound molecules, which remains around 2.8 l/d-Tyr per nanocluster with 10-analyte runs (*P* = 28%) and 4.7/4.3 l/d-Tyr per nanocluster with 20-analyte runs (*P* = 22%).

Similar to the 38*p*MBA nanocluster, the 102*p*MBA nanocluster displays moderate (*P* = 21.4%) to very strong (*P* = 100%) interactions with l-GSH, l/d-Arg, l/d-Cys, l/d-Tyr, l/d-Ser, and l/d-Ala. Interestingly, we observed enantioselectivity in five cases, with stronger interactions to l-GSH, d-Cys, d-Tyr, l-Ser, and l-Ala as compared to their enantiomers. Figures S9–S11 show the data for time variations of the COM distance, HB number, Coulomb energy, and LJ energy, followed by a detailed discussion of the selected bound structures in Figure S12. We did not study concentration effects of analytes for 102*p*MBA, nor for 144*p*MBA that will be discussed next.

The 144*p*MBA nanocluster shows strong interactions only with a single l/d-Arg (*P* ≈ 98%), with the COM data and selected bound structures shown in Figure S13, followed by a detailed discussion in the Supporting Information. However, it is interesting to note that the modes of interaction of l/d-Arg with 144*p*MBA are different from the cases of 38*p*MBA and 102*p*MBA, in the sense that we do not observe the analyte penetrating the ligand shell of 144*p*MBA. We hypothesize that the reason is the increased packing density of ligands in 144*p*MBA due to the smaller curvature of its gold–thiolate interface. Indeed, a simple spherical model predicts that the number of ligands on a gold nanocluster with *N* gold atoms should increase by a power of 2/3 with respect to the number of ligands in a smaller cluster of *N’* gold atoms, if the ligand density remains constant. For the two largest *p*MBA nanoclusters, one expects an increase in the number of ligands as (144/102)^2/3^ = 1.25; however, the actual ligand ratio is 60/44 = 1.36. This shows that, once the curvature of the nanocluster surface gets smaller, the ligand density increases toward a limiting value observed on flat, self-assembled thiolate overlayers on the Au(111) planar surface.

### 2.4 Circular Dichroism and the Sensing Potential

Summarizing the above discussion on the chemical and enantiospecific selectivity of nanocluster–analyte interactions, we noted that, while both GSH- and *p*MBA-protected nanoclusters interact strongly with arginine, the *p*MBA nanoclusters offer more versatile behavior that could be utilized for sensor design. 144*p*MBA binds strongly only with Arginine, while 38*p*MBA and 102*p*MBA have moderate to strong interactions with a larger group of analytes, and 102*p*MBA offers the best potential for enantioselective binding. Enantioselective binding could lead to experimentally detectable changes in the CD signal of the gold nanocluster. This hypothesis motivated us to perform linear-response time-dependent DFT calculations of a number of nanocluster–analyte complexes, as shown in Figures 4 and 5. As the calculations were numerically intensive, we had to concentrate on studying only the smallest 25GSH, 38GSH, and 38*p*MBA nanoclusters with a subset of analytes.

**Figure 4:**
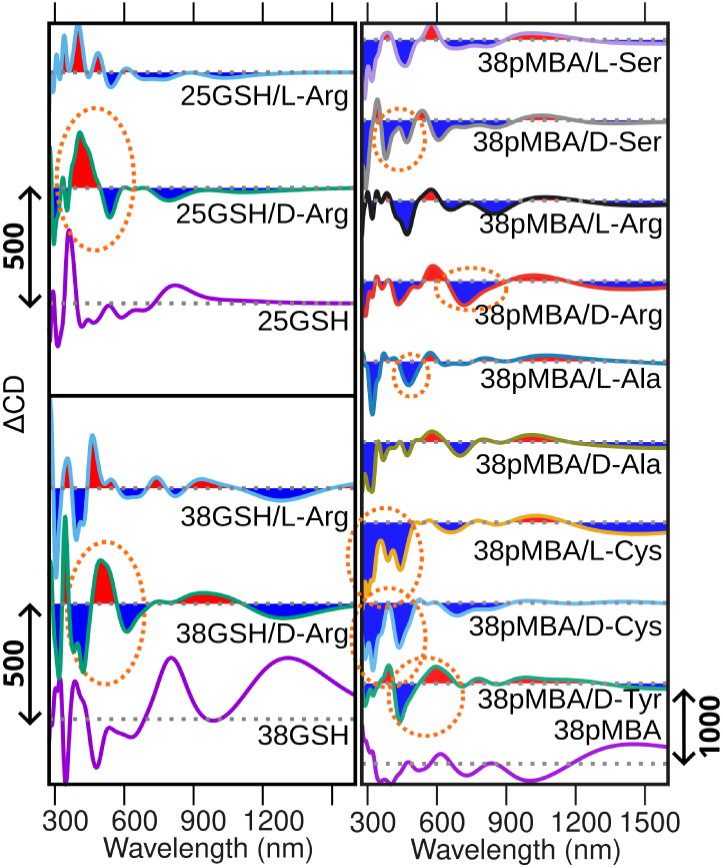
Calculated difference CD spectra of 25GSH, 38GSH, and 38*p*MBA nanoclusters with single analytes. The difference spectra are calculated using statistically averaged spectra, showing positive (red) and negative (blue) contributions with respect to the reference spectrum of the bare nanocluster without analytes, shown at the bottom of each panel. The intensity scales are denoted by arrows in each panel. Dashed ellipses highlight the main identifiable features of the CD signal in the nanocluster–analyte hybrids.

**Figure 5:**
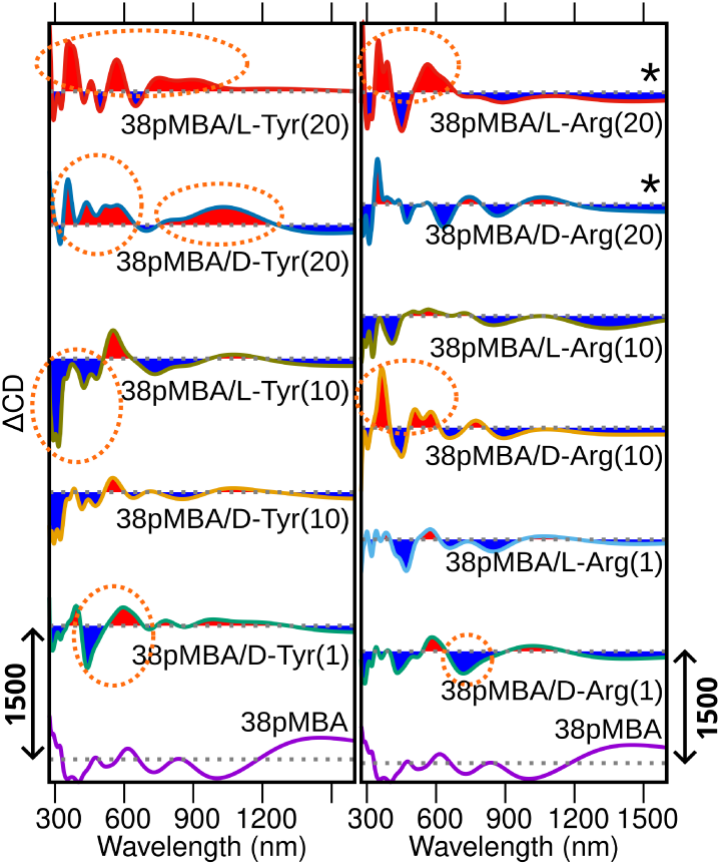
Calculated difference CD spectra of 25GSH, 38GSH, and 38*p*MBA nanoclusters with multiple analytes. The difference spectra are calculated using the statistically averaged spectra, showing positive (in red) and negative (in blue) contributions with respect to the reference spectrum of the bare nanocluster without analytes, shown at the bottom of each panel. The intensity scales are denoted by arrows in each panel. Dashed ellipses highlight the main identifiable features of the CD signal in the nanocluster–analyte hybrids. 38*p*MBA/l-Arg­(20) and 38*p*MBA/d-Arg­(20) have a reduced number of snapshots in statistical sampling due to computational limitations.

Figures 4 and 5 show that, in general, the analyte binding can induce changes in the nanoclusters’ CD spectrum across a wide range from the UV to NIR wavelengths, with the strongest variations in the region of 300–600 nm (see statistically averaged CD spectra in Figure S14). Previous theoretical studies of the chiral response of 25GSH and 38*p*MBA nanoclusters have assigned signals in this region mostly to the thiol–gold interface and the ligands.^22^ Hence, chiral analyte binding can be expected to have the strongest impact in this region as well.

We noted that, from the GSH-protected nanoclusters, 25GSH has potential for enantiospecific sensing of l/d-Arg, since both enantiomers bind rather strongly and the handedness of the analyte affects the differential CD spectrum around 400 nm. 38GSH nanoclusters can detect the binding of l/d-Arg but not the handedness of the analyte. 38*p*MBA nanoclusters can detect the binding of single d-Tyr, l/d-Cys, l/d-Ala, and l/d-Ser, with potential for enantiospecific sensing of analytes other than cysteine. Furthermore, 38*p*MBA nanoclusters cannot sense the enantiospecific binding of one l/d-Arg, but interestingly, the sensitivity increases for higher concentrations of the analyte. Higher concentrations of the analyte also increase the enantiospecific sensing for l/d-Tyr. Based on the calculated changes in the CD signal, we summarize the sensing potential of these systems in Table 2.

**Table 2:** Potential for Enantiospecific Sensing^a^

Sensing potential	*P*(1) %	SE(1)	SB(10)	SE(10)	SB(20)	SE(20)
Complex						
25GSH/l-Arg	73.5	Y	-	-	-	-
25GSH/d-Arg	77.2	Y	-	-	-	-
38GSH/l-Arg	64.5	N	-	-	-	-
38GSH/d-Arg	71.9	N	-	-	-	-
38*p*MBA/l-Arg	100	N	Y	Y	Y	Y
38*p*MBA/d-Arg	100	N	Y	Y	Y	Y
38*p*MBA/l-Tyr	3.3	-	Y	N	Y	Y
38*p*MBA/d-Tyr	53.3	Y	Y	N	Y	Y
38*p*MBA/l-Cys	56.8	N	-	-	-	-
38*p*MBA/d-Cys	46.3	N	-	-	-	-
38*p*MBA/l-Ala	27.6	Y	-	-	-	-
38*p*MBA/d-Ala	26.7	Y	-	-	-	-
38*p*MBA/l-Ser	33.3	Y	-	-	-	-
38*p*MBA/d-Ser	36.7	Y	-	-	-	-

a *P*(1) represents the probability (in %) of a single analyte binding, as reproduced from Table 1. The potential for enantiospecific sensing of one analyte, SE(1), along with the potential for sensing the binding at higher concentrations, SB(10) and SB(20), and enantiospecific sensing at higher concentrations, SE(10) and SE(20), are qualitatively indicated with labels “Y” (yes) or “N” (no).

## 3 Conclusions

We have evaluated the potential of ligand-protected gold nanoclusters as noninvasive chiral sensors of small chiral biomolecules in aqueous media by performing an extensive computational screening of 96 chiral nanocluster–chiral analyte complexes in water. We found promising variations in the sensing ability of the nanoclusters, as controlled by their ligand design and the size of the metal core, from 25 to 144 gold atoms. The variations discriminate different nanocluster–analyte interactions inside the ligand shell or at the gold–ligand interface, impacted by hydrogen bonding, electrostatic interactions, van der Waals interactions, or the available steric volume controlled by the curvature of the gold–ligand interface. Due to the widely available synthetic strategies to precisely control the nanocluster geometry, size, ligand-shell chemistry, and even preferred chirality^23,36^ for 1–2 nm gold nanoclusters, we expect that the development of a toolbox of water-soluble gold nanoclusters in this size range, tuned to sensitive detection of a given set of small chiral biomolecules, should be an experimentally realizable concept. Accompanied by numerical or machine-learning algorithms for the fingerprint and shape detection of the differential CD spectra, this method could become highly accurate and sensitive for all analytes and enantiomers. This strategy could be complemented by other size- and structure-sensitive experimental methods, such as diffusion-ordered spectroscopy nuclear magnetic resonance (DOSY-NMR) of nanocluster–analyte hybrids.

## 4 Methods

### 4.1 Classical MD Simulations

The atomic structures of the six thiolate-protected AuNCs were derived from reported experimental structures of same-size nanoclusters, but with phenylethyl thiolate ligands for Au_25_ and Au_38_ (refs 37,38 ) and phenylmethyl thiolate ligands for Au_144_ (ref 39). The structure of Au_102_(*p*MBA)_44_ was taken from the crystal structure reported by the Kornberg group.^40^ Starting from these initial structures, ligand substitution followed by DFT optimization was carried out, providing the starting geometries for the MD simulations. The structures of the L forms of the analytes were taken from the literature,^41^ while the corresponding d enantiomers were built using Maestro software.^42^ The protonation states of *p*MBA and GSH ligands, as well as analyte side chains, were adjusted at pH 7 by using the charges of functional groups based on literature p*K*_a_ values. All MD simulations were performed using the GROMACS package (version 2024.3)^43^ and our previously published AMBER-compatible force field for thiolate-protected AuNCs,^44^ together with AMBER parameters for zwitterionic amino acids.^41^ These AuNC parameters have been successfully applied in earlier studies on enterovirus–AuNC interactions^45^ and in the design of AuNCs for targeted drug delivery in gastric cancer therapy.^46^

All simulations were conducted in a cubic simulation box of approximately 11.5 × 11.5 × 11.5 nm^3^, with periodic boundary conditions applied in all three dimensions. Depending on the system, approximately 45,000–50,000 water molecules, described by the TIP3P water model,^47^ were added. The negatively charged ligand layer of the AuNCs was neutralized by the addition of Na^+^ counterions. No additional counterions were used. The corresponding ionic strengths vary from 0.014 to 0.079 M, depending on the cluster size. Each system was first energy-minimized using the steepest-descent algorithm, followed by equilibration in the NVT ensemble at 300 K for 10 ns using a velocity-rescaling thermostat, and subsequently in the NPT ensemble at 1 bar for an additional 10 ns using the C-rescale barostat. During equilibration, positional restraints were applied to the AuNC and analyte. Production simulations were then performed in the NPT ensemble without any positional restraint for 500 ns. A time step of 2 fs was used throughout. The electrostatic interactions were treated using the Particle Mesh Ewald (PME) method with a cutoff of 1.0 nm, and the van der Waals interactions were modeled with Lennard-Jones potentials.

To improve the statistical sampling of AuNC–analyte interactions, each system was simulated using three independent MD replicas initiated with different atomic velocities. In total, 288 single-analyte systems were simulated, corresponding to 48 simulations per AuNC (3 replicas × 2 enantiomers × 8 analytes). The first 100 ns of each trajectory were discarded as equilibration. Trajectory analysis was carried out using GROMACS utilities, and visualization was performed using VMD.^48^

Several metrics were employed to characterize AuNC–analyte interactions, including the distance between the center of mass (COM) of the AuNC and the analyte, the number of hydrogen bonds (HB) between them, and the Coulombic (Coul) and Lennard–Jones (LJ) interaction energies. In addition, the number of contacts (NOC) between the AuNC and the analyte was defined as the number of atom pairs within 0.6 nm between heavy atoms (i.e., excluding hydrogen atoms) of the analyte and the AuNC. Based on the NOC values obtained from three replicas, an adsorption probability, *P*, was defined as

$$P=\frac{\underset{\mathrm{NOC}>5}{\sum }N}{N_{\mathrm{total}}}$$

where *N* is the number of frames in which the NOC exceeds five, and *N*_total_ is the total number of frames. A value of *P* = 1 thus indicates continuous adsorption throughout all replicas.

The effect of analyte concentration was investigated by increasing the number of analyte molecules to 10 or 20 in a larger cubic simulation box of approximately 14 × 14 × 14 nm^3^, containing about 90,000 water molecules, with ionic strengths for Na+ counterions varying from 0.008 to 0.044 M. The simulated analyte concentration are 109, 605, and 1210 μM for systems containing 1, 10, or 20 analytes, respectively. Although these concentrations exceed those reported *in vivo* for D-amino acids in cancer patients and healthy subjects,^12,13^ the use of elevated concentrations facilitates observation of adsorption events within accessible simulation time scales. Each high-concentration system was simulated for 1 μs, with all other simulation parameters identical to those used in single-analyte simulations. Concentration effects were investigated for Au_38_(GSH)_24_ interacting with l- and d-Arg, and for Au_38_(*p*MBA)_24_ interacting with l- and d-Arg and l- and d-Tyr. This resulted in 12 additional simulations for Au_38_(GSH)_24_ (3 replicas × 4 systems) and 24 for Au_38_(*p*MBA)_24_ (3 replicas x 8 systems). The number of adsorbed analytes was estimated by counting contacts between the inner Au atoms of the AuNC core and the C_α_ atom of the analyte (Arg or Tyr) within a cutoff distance of 1.5–2.5 nm, adjusted depending on the analyte and nanocluster size.

Structural heterogeneity across the MD trajectories was assessed using the final 400 ns of each replica (1.2 μs in total). Representative structures of adsorbed analytes were identified via RMSD-based geometric clustering of the analyte C_α_ atom and the heavy atoms of the AuNC, using cutoff values between 0.6 and 1.5 Å. Cluster centers corresponding to the largest clusters with the smallest average RMSD were selected as representative configurations.

Finally, principal component analysis (PCA)^49,50^ was performed on the heavy atoms of the AuNCs during analyte interaction over the final 400 ns of each trajectory. Projection of the trajectories onto the first two principal components enabled identification of configurations located within the minimum Gibbs free energy regions. These frames were subsequently subjected to RMSD analysis and selected for DFT-based CD calculations, as described in Section 4.3. For simulations involving higher analyte concentrations, PCA was performed over the final 900 ns of each replica.

### 4.2 Statistical Sampling

The procedure used to select representative MD snapshots for the statistical sampling of CD spectra of nanocluster–analyte hybrids and bare nanoclusters is illustrated in Figure S15. The approach combines PCA, conformational free-energy profiling, and RMSD analysis to identify the most relevant configurations. PCA was performed following the methodology described in ref 22. The numerical details of the sampling procedure and the selected configurations are given in Table S1.

### 4.3 DFT Calculations of the CD Spectra

CD spectra were computed using time-dependent density functional theory (TDDFT) as implemented in the GPAW code.^51^ The ground-state calculations were done using real-space grids with a spacing of 0.25 Å, employing an implicit water solvent model and the GLLB-sc exchange-correlation (xc) functional^52^ for the description of the wave functions. CD spectra were obtained by using the linear-response formalism of TDDFT,^53^ with the GLLB-sc wave functions as the basis and the PBE functional^54^ as the xc-kernel.

For nanocluster–analyte hybrids, only analyte molecules with a minimum atom–atom distance of less than 4.0 Å from the nanocluster were included in the DFT calculations. The CD spectra of individual nanocluster–analyte configurations were weighted according to the Boltzmann factor derived from the conformational free energies of the representative structures selected through the statistical sampling procedure described above. The resulting weighted-average CD spectrum of each nanocluster–analyte hybrid was compared with the correspondingly weighted-average spectrum of the bare nanocluster. Conclusions regarding the sensing ability were drawn from the calculated difference spectra (Figures 4 and 5).

## Supplementary Material



## Data Availability

Raw data from cluster-analyte molecular dynamics simulations are available by request to authors.

## Notes

Additional discussion, Figures S1–15, and Table S1 (PDF)
