# Supplementary material for: Chiral Ligand-Protected Gold Nanoclusters as Biosensors for Small Chiral Biomolecules: A Computational Study
Source: ACS Nano. 2026 Feb 25;20(9):7743–51. doi: 10.1021/acsnano.5c20222 (PMC12981017; doi:10.1021/acsnano.5c20222)
Supplement: Supplementary file 1 [file nn5c20222_si_001.pdf]

Supporting Information for

Chiral ligand-protected gold nanoclusters as biosensors to small chiral biomolecules: A Computational study

Zohreh Fallah[1], Sami Malola[1], María Francisca Matus[1] and Hannu Häkkinen[1,2,*]

[1]Department of Physics, Nanoscience Center, University of Jyväskylä, FI-40014 Jyväskylä, Finland

[2]Department of Chemistry, Nanoscience Center, University of Jyväskylä, FI-40014 Jyväskylä, Finland

Corresponding author email: hannu.j.hakkinen@jyu.fi

*p*MBA-protected nanoclusters with analytes

38*p*MBA/Arg

Representative binding configurations of Au_{38}(*p*MBA)$_{24}$ with L- and D-Arg are shown in Fig. S7(a-e). These structures illustrate distinct adsorption modes depending on the orientation of the Arg functional groups. In one mode, the amino group of Arg interacts with the AuNC core region, involving Au atoms and the S atoms of *p*MBA, while the guanidinium group forms interactions with the carboxylate group of *p*MBA; this configuration is observed for L-Arg in Fig. S7(a) and for D-Arg in Fig. S7(d). In an alternative arrangement, the guanidinium group interacts with the core region of the AuNC, whereas the amino group engages with the *p*MBA carboxylate (L-Arg in Fig. S7(b) and D-Arg in Fig. S7(e)). A deeper adsorption mode is also observed, in which both the amino and guanidinium groups of Arg interact simultaneously with the nanocluster core. This configuration occurs between approximately 257-363 ns in the third replica of the L-Arg simulation and is associated with the smallest COM (~0.75 nm) between L-Arg and Au_{38}(*p*MBA)$_{24}$, as indicated by the arrow in Fig. S4(a). This deeper interaction correlates with a reduced number of HB between the AuNC and L-Arg (Fig. S5(a)) and diminished fluctuations in the Coulombic interaction energy (Fig. S5(b)). The corresponding deeply adsorbed configuration is shown in Fig. S7(c). Overall, Arg exhibits deeper penetration into the core region of Au_{38}(*p*MBA)$_{24}$ nanoclusters compared to GSH-protected nanoclusters, where adsorption predominantly occurs at the outer surface of the ligand layer.

38*p*MBA/Tyr

As discussed in the main text, Au_{38}(*p*MBA)$_{24}$ exhibits enantioselective interactions with Tyr. As shown in Figs. S4(c,d), D-Tyr remains adsorbed in the nanocluster throughout the entire MD trajectory in the second replica, whereas in the first and third replicas it undergoes repeated adsorption and desorption events. D-Tyr primarily interacts via its amino groups, adopting different orientations that allow penetration into the core region of Au_{38}(*p*MBA)$_{24}$. The number of HB between D-Tyr and Au_{38}(*p*MBA)$_{24}$ (Fig. S5(j)) indicates that HB does not play a dominant role in adsorption. Instead, LJ interactions dominate over Coulombic contributions (Figs. S5(k,l)), likely due to pi-pi stacking between p-hydroxyphenyl side chain of Tyr and benzoic acid moiety of *p*MBA. Representative snapshots of D-Tyr adsorption at t=325.7 ns (second replica; Fig. S7(f)) and t=195.6 ns (third replica; Fig. S7(g)) illustrate configurations associated with distinct LJ energies (Fig. S5(l)), which correlate with small differences in the COM distance from the nanocluster core (Fig. S4(d), arrows).

38*p*MBA/Cys

L- and D-Cys adsorb at different regions of Au_{38}(*p*MBA)$_{24}$. In the dominant adsorption mode, the Cys sulfhydryl group interacts with the carboxyl group of *p*MBA, while the amino group

penetrates toward the nanocluster core (Au atoms and S atoms of *p*MBA), as shown in Fig. S7(h) for L-Cys and Fig. S7(i) for D-Cys. A less deeply adsorbed configuration of D-Cys is observed at t=202.3 ns in the first replica (Fig. S7(j)), characterized by a larger COM distance from the core. Energy analyses (Figs. S5(n,o,q,r)) reveal that L-Cys exhibits similar LJ and Coul interaction energies across replicas, whereas D-Cys shows two distinct energetic regimes. One corresponds to deep, persistent adsorption throughout the entire 500 ns trajectory (second replica), with energies comparable to L-Cys, while the other corresponds to weaker, less stable adsorption (Fig. S7(j)). These differences are reflected in the COM distance distributions (Fig. S4(f)). Overall, L-Cys exhibits a higher adsorption probability than D-Cys, indicating that deeper penetration into the core region plays a key role in stabilizing adsorption.

38*p*MBA/Ser

Both L- and D-serine adsorb on Au_{38}(*p*MBA)$_{24}$ via their amino groups interacting with the core region. Differences in adsorption depth are reflected in distinct LJ interaction energies (Figs. S6(c,f)), similar to the behavior observed for Tyr and Cys. COM distance analyses (Figs. S4(g,h)) confirm that variations in LJ energy arise from more or less pronounced penetration into the core region. The snapshot of L-Ser at t=293 ns in the second replica (Fig. S7(k)) corresponds to the most negative LJ energy and the deepest adsorption. A second representative configuration at t=178.4 ns (Fig. S7(l)) shows adsorption at the same site but with a slightly larger core distance and weaker LJ interaction (Fig. S6(c)), attributable to a different orientation of the serine hydroxyl group relative to the core.

38*p*MBA/Ala

For alanine, Coulombic interaction energies show minimal variation upon adsorption for both enantiomers (Figs. S6(h,k)), whereas LJ interactions adopt discrete values depending on adsorption depth. COM distance analyses (Figs. S4(i,j)) indicate that these LJ variations correspond to different extents of amino-group penetration into the core region. Representative structures illustrate this behavior: L-Ala at t=352.4 ns (third replica; Fig. S7(n)) and t=134.1 ns (second replica; Fig. S7(o)) correspond to shallow and deep adsorption modes, respectively. Similarly, D-Ala at t=423.8 ns (second replica; Fig. S7(p)) represents a deeply adsorbed state with a more negative LJ energy (Fig. S6(l)), whereas a shallower configuration at t=331.2 ns (Fig. S7(q)) exhibits weaker LJ interactions.

38*p*MBA/Arg(10)

At higher analyte concentration, arginine molecules exhibit adsorption behavior similar to that observed for single-molecule simulations, with frequent penetration into the core region while

interacting with the ligand layer. The increased number of adsorbed Arg molecules enables identification of preferred binding motifs. Most L- and D-Arg molecules interact with the nanocluster via their guanidinium groups (Figs. S8(e–h)). As shown in Figs. S8(g,h), intermolecular Arg–Arg interactions appear to promote cooperative adsorption, consistent with observations for $Au_{38}(GSH)_{24}$.

38*p*MBA/Tyr(10)

As shown in Figs. S8 (i) and (j), on one side, at least one D-Tyr adsorb on $Au_{38}(p\text{MBA})_{24}$ during the whole 1000 ns in all three replicas in 10 D-Tyr system, wherein the maximum number of adsorbed D-Tyr can be 6. On the other side, although maximum 6 (out of 10) L-Tyr molecules can adsorb simultaneously on AuNC, it happens within the first 324 ns of the second replica that none of 10 L-Tyr molecules adsorbs on AuNCs. This is in agreement with single L- and D-Tyr simulation, where only D-Tyr adsorb on $Au_{38}(p\text{MBA})_{24}$. As shown in Figs. S8(k,l), by increasing the number of L- or D-Tyr molecules to 20, at least one L- or D-Tyr adsorbs on AuNC while maximum 9 (out of 20) of L- or D-Tyr molecules can adsorb simultaneously on AuNC. As shown in some snapshots in Figs. S8(m-p), it can also be seen that the amino group of one Tyr can interact with p-hydroxy phenyl methyl side chain of another Tyr molecule which could improve the adsorption of Tyr molecules on AuNC. Therefore, Tyr-Tyr interaction including pi-pi stacking between p-hydroxy phenyl methyl side chain of Tyr could improve the adsorption of L or D-Tyr molecules on AuNC.

102*p*MBA/Arg

Both L- and D-Arg interacts within the core area and carboxyl group of ligand layer. The snapshot of L-Arg at t=201.5 ns in the first replica and at t=191.4 ns in the third replica and D-Arg at t=456.6 ns in the third replica as well as at t=385.8 ns in the first replica have been shown in Figs. S12(a-d), respectively. The guanidine group of L-Arg in the third replica interacts mainly with the core area, so the amino group interacts with the carboxyl group of *p*MBA and also the surrounding water molecules. It is while in the first and second replicas, L-Arg interacts mainly with its amino group with the core area and the side chain interacts with the carboxyl group of *p*MBA and the aqueous environment. As mentioned, similar behaviour has been observed when D-Arg adsorbs on $Au_{102}(p\text{MBA})_{44}$ but as it can be seen in Fig. S9(b), the COM distance between D-Arg and AuNC in the third replica is a little more than other two replica MDs. D-Arg interacts with its amino group with the core area in the first replica while in the second and third replicas it interacts with its guanidine group with the core area. But while the guanidine group interacts with the core area in both replicas (2 and 3), D-Arg another tail (amino and carboxyl group) interacts with the surface of *p*MBA in replica 2. It means D-Arg bends toward the ligand layers, but in the third replica, other tail of D-Arg stays straight and

only interact with the surrounding water molecules. This is why the COM distance of D-Arg in the third replica is more than the second replica.

102*p*MBA/Cys

There is a considerable difference between L and D-Cys when they adsorb on Au_{102}(*p*MBA)$_{44}$, as D-Cys has a P of 100% and adsorbs on AuNC on the whole MD time (1500 ns) but L-Cys with P of 47.43% has almost the half adsorption probability of D-Cys onto Au_{102}(*p*MBA)$_{44}$, this behaviour has not been seen between L- and D-Cys in other AuNCs. The number of HB in Fig. S10(a,d) show that HB does not play an important role in adsorption of Cys on AuNC, as there is no HB between D-Cys and Au_{102}(*p*MBA)$_{44}$ on three replicas and no HB between L-Cys and AuNC in the second replica where L-Cys adsorbs on Au_{102}(*p*MBA)$_{44}$. The same behaviour has been observed in adsorption of L- and D-Cys on Au_{38}(*p*MBA)$_{24}$. D-Cys interacts deeply with its amino group with the core area while its sulfhydryl group interacts with pMBA, but L-Cys has the same deep interaction with the core area only in the second replica (representative L-Cys at t=412.4 ns in Fig. S12(e)) and less deep interaction in a part of the third replica (representative L-Cys at t=403 ns in Fig. S9(f)) which shows less negative Coul and LJ energy (Figs. S10(b,c,e,f)). A little more COM distance between L-Cys and AuNC in part of the third replica than the second replica is observable in Fig. S9(c). Central structure of D-Cys in the largest geometric cluster at t=191.1 ns in the third replica is shown in Fig. S12(g).

102*p*MBA/Ala

As discussed, there is a significant difference between adsorption of L- and D-Ala on Au_{102}(*p*MBA)$_{44}$, as L-Ala adsorbs on AuNC on the whole time of three replicas (P=100%) while D-Ala absorbs much less on AuNC (P=36.38%). As shown in Figs. S10(g,j), although there is HB between L- or D-Ala and Au_{102}(*p*MBA)$_{44}$, it does not happen on all analyte-adsorbed MD frames. Similar to Au_{38}(*p*MBA)$_{24}$, Ala in both L- and D-forms interacts with its amino group with the core area of Au_{102}(*p*MBA)$_{44}$, as it can be seen in the representative structures of L- and D-Ala in Figs. S12(h-j). Figs. S10(h,i,k,l) show that LJ energy is stronger than Coul energy in adsorption of Ala on Au_{102}(*p*MBA)$_{44}$ which seems logical based on hydrophobic side chain of Ala. D-Ala has the same Coul energy with AuNC when adsorbs on Au_{102}(*p*MBA)$_{44}$ while the LJ energy with AuNC in the second replica is less negative (Fig. S12(j)) than the LJ energy in the first replica (Fig. S12(i)), which corresponds to more deep interaction with the core area in the first replica, as can be seen in Fig. S9(f).

102*p*MBA/Tyr

Tyr in both L- and D-forms has the best adsorption on Au_{102}(*p*MBA)$_{44}$ among all investigated AuNCs. D-Tyr with P of 61.09% adsorbs more than L-Tyr with P of 29.97% on Au_{102}(*p*MBA)$_{44}$. Unlike Au_{38}(*p*MBA)$_{24}$, L-Tyr with P of 29.97% has some adsorption on Au_{102}(*p*MBA)$_{44}$. Due to deeper interaction of the analyte amino group with the core area, number of HB happening between analyte amino group with carboxyl group of *p*MBA is less probable, it is why number of HB between D-Tyr and Au_{102}(*p*MBA)$_{44}$ is almost zero (Fig. S10(p)). The LJ energy between L-Tyr and AuNC has different values based on a little less or deeper adsorption within the core area, while D-Tyr has the same LJ energy value when adsorbs on AuNC. The representative structures of L- and D-Tyr in Figs. S12(k,l), respectively, showing that L- and D-Tyr amino groups interact with the core area while it seems there is stacking interaction between p-hydroxy phenyl methyl side chain of Tyr and benzoic acid of *p*MBA.

102*p*MBA/Ser

L-Ser with P of 34.93% adsorbs more on Au_{102}(*p*MBA)$_{44}$ than D-Ser with P of 21.42%. As shown in Figs. S11(a,d), there are HB between both L- and D-Ser and Au_{102}(*p*MBA)$_{44}$ when interacting with AuNC which could be due to the hydroxyl group of Ser side chain, even when Ser interacts with its amino group deeply with the core area of AuNC. Representative structures of L-Ser at t=335.7 ns of the first replica, and D-Ser at t=304.6 ns of the third replica with Au_{102}(*p*MBA)$_{44}$ in Figs. S12(m,n), respectively, demonstrate the adsorption of Ser amino group within the core area of AuNC.

102*p*MBA/GSH

Au_{102}(*p*MBA)$_{44}$ is the only AuNC that glutathione as an analyte adsorbs on. As it can be seen in COM plots in Figs. S9(k,l), D-GSH does not adsorb on AuNC while L-GSH in one of three replica MDs (replica 3) adsorbs on Au_{102}(*p*MBA)$_{44}$ in the whole 500 ns MD. Therefore, as it happens only in one of the replicas, the P value is 33.73% (Table 1). As shown in Fig. S11(g), HB plays an important role in adsorption of L-GSH on Au_{102}(*p*MBA)$_{44}$. The representative structure of L-GSH at t=284 ns of the third replica with AuNC has been shown in Fig. S12(o). The Coul and LJ between L-GSH and Au_{102}(*p*MBA)$_{44}$ during three replica MDs have been plotted in Figs. S11(h) and (i) show that Coul energy between L-GSH and AuNC is a little stronger than the corresponded LJ energy, which is expectable, as GSH is negatively charged at pH of 7. As shown in Fig. S12(o), GSH interacts with the amino group (of glutamate) with the core area of Au_{102}(*p*MBA)$_{44}$ while sulfhydryl group and NH of carboxamide group in Cys part interact with the benzoic acid of *p*MBA.

144*p*MBA/Arg

L- and D-Arg interact with their amino and guanidine group with the carboxyl group of *p*MBA. It means unlike other two *p*MBA-protected nanoclusters (Au_{38} and Au_{102}), Arg does not reside between the ligands and does not interact within the core area, as shown in the representative central cluster structures of L- and D-Arg in Figs. S13(c-f). The guanidine and amino groups of L/D-Arg interact with the carboxyl group of *p*MBA and do not interact with the core area. L- and D-Arg interact with different ligands of AuNC. It means somehow, they walk on the surface of ligand layer, and sometimes leave the AuNC and then adsorb again, it is why the COM distance in Figs. S13(a) and (b) fluctuate more.

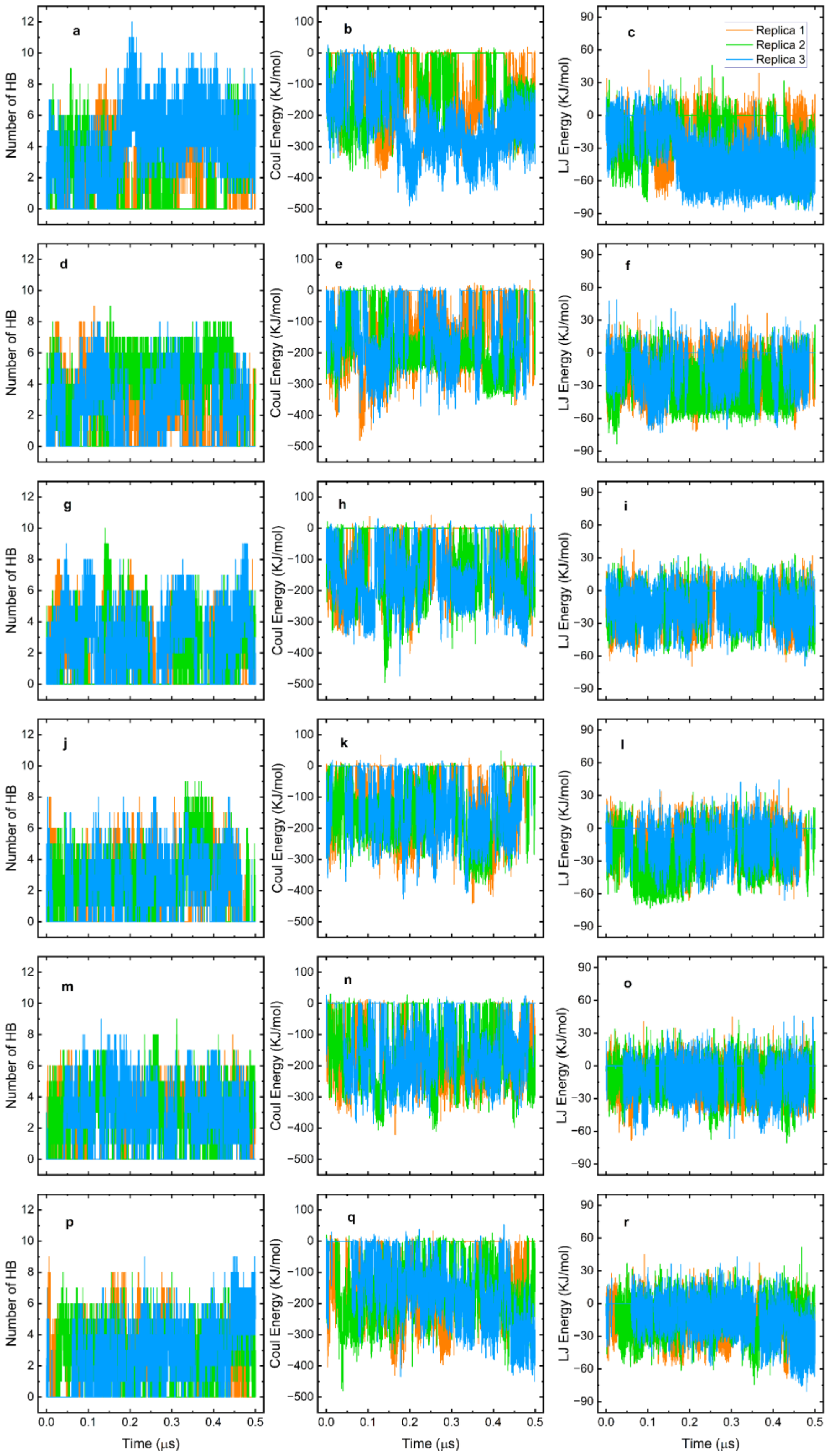

Fig. S1. Arginine as analyte. (a) The number of HB between L-Arg and $Au_{25}(GSH)_{18}$, (b) the Coul and (c) LJ energy between L-Arg and $Au_{25}(GSH)_{18}$, (d) the number of HB between D-Arg and $Au_{25}(GSH)_{18}$, (e) the Coul and (f) LJ energy between D-Arg and $Au_{25}(GSH)_{18}$, (g) the number of HB between L-Arg and $Au_{38}(GSH)_{24}$, (h) the Coul

and (i) LJ energy between L-Arg and $Au_{38}(GSH)_{24}$, (j) the number of HB between D-Arg and $Au_{38}(GSH)_{24}$, (k) the Coul and (l) LJ energy between D-Arg and $Au_{38}(GSH)_{24}$, (m) the number of HB between L-Arg and $Au_{102}(GSH)_{44}$, (n) the Coul and (o) LJ energy between L-Arg and $Au_{102}(GSH)_{44}$, (p) the number of HB between D-Arg and $Au_{102}(GSH)_{44}$, (q) the Coul and (r) LJ energy between D-Arg and $Au_{102}(GSH)_{44}$ in three independent MD replicas.

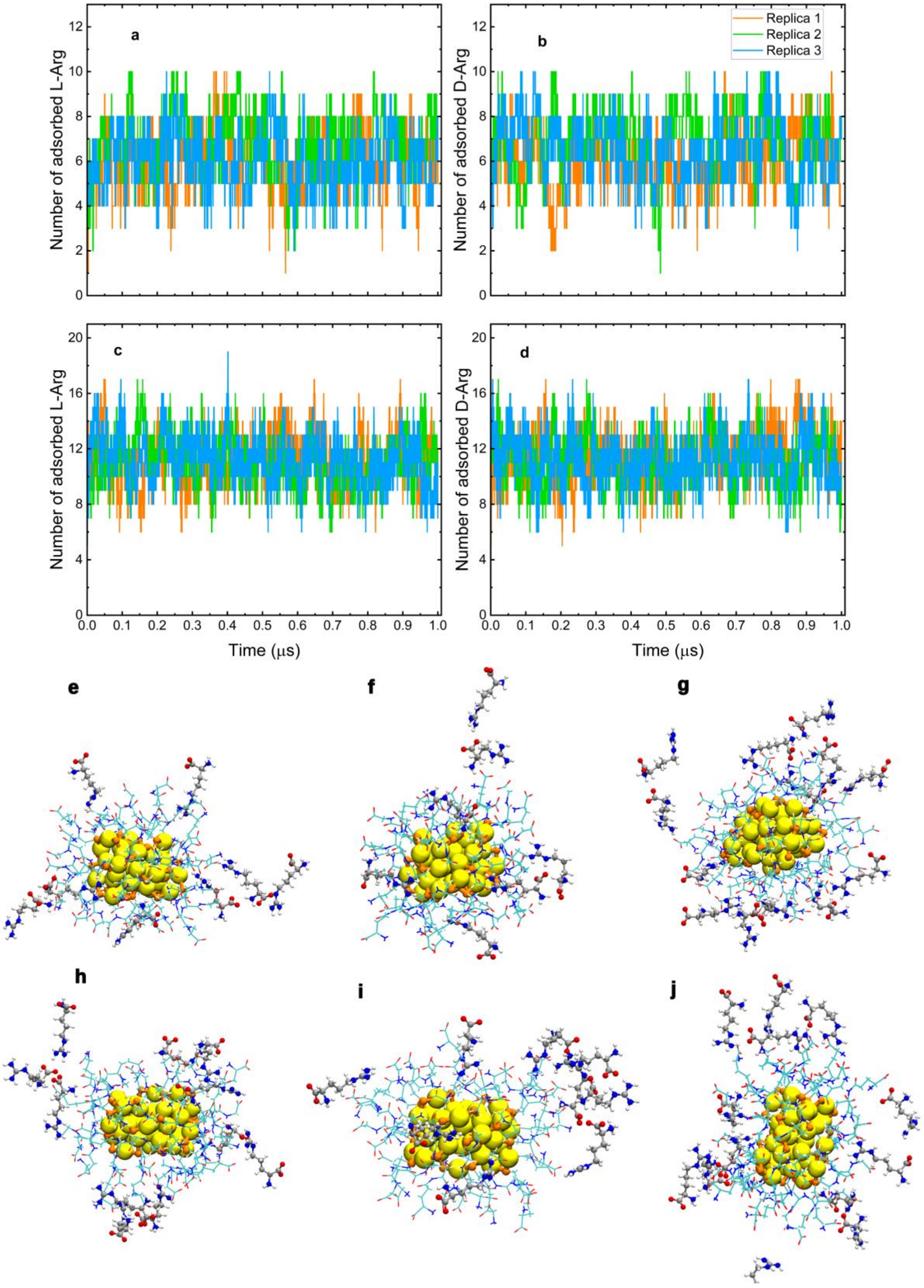

Fig. S2. Adsorbed L-/D-Arg molecules on $Au_{38}(GSH)_{24}$. Number of bound molecules on $Au_{38}(GSH)_{24}$ in (a) 10 L-Arg, (b) 10 D-Arg, (c) 20 L-Arg and (d) 20 D-Arg MD runs. $Au_{38}(GSH)_{24}$ with (e) 10 L-Arg molecules in the second replica at t=478 ns, (f) 10 D-Arg molecules in the first replica at t=668 ns, (g) 20 L-Arg molecules in the second replica at t=861 ns, (h) 20 D-Arg molecules in the first replica at t=497 ns, (i) 20 L-Arg molecules in the first replica

at t=397 ns, and (j) 20 D-Arg molecules in the third replica at t=480 ns. Colour code: Au=yellow; S=orange; O=red; N=blue; H=white; C (ligand layer)=cyan; C (analyte)=silver.

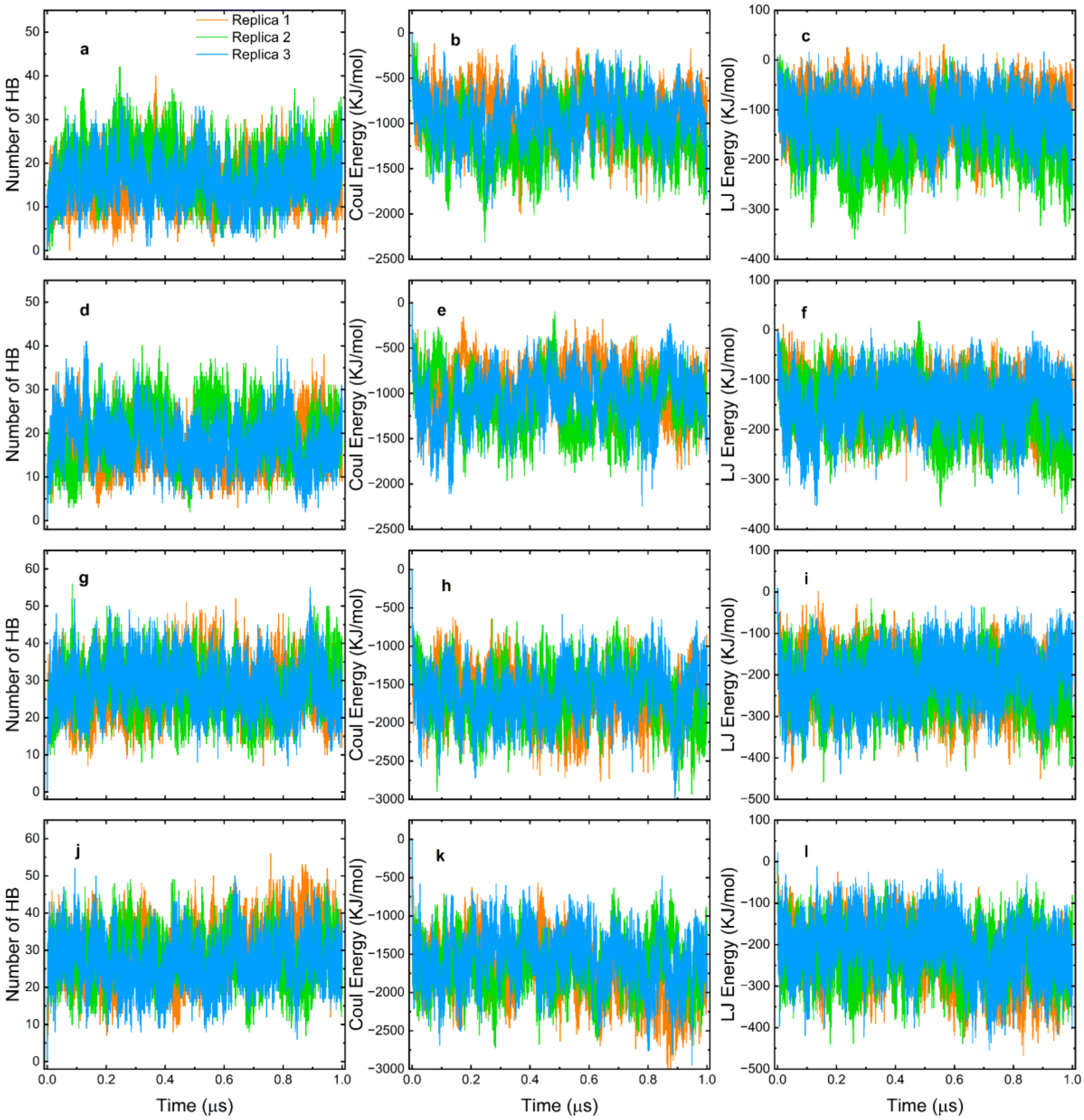

Fig. S3. Arginine as analyte. (a) The number of HB between 10 L-Arg and $Au_{38}(GSH)_{24}$, (b) the Coul and (c) LJ energy between 10 L-Arg and $Au_{38}(GSH)_{24}$, (d) the number of HB between 10 D-Arg and $Au_{38}(GSH)_{244}$, (e) the Coul and (f) LJ energy between 10 D-Arg and $Au_{38}(GSH)_{24}$, (g) the number of HB between 20 L-Arg and $Au_{38}(GSH)_{24}$, (h) the Coul and (i) LJ energy between 20 L-Arg and $Au_{38}(GSH)_{24}$, (j) the number of HB between 20 D-Arg and $Au_{38}(GSH)_{24}$, (k) the Coul and (l) LJ energy between 20 D-Arg and $Au_{38}(GSH)_{24}$ in three independent MD replicas.

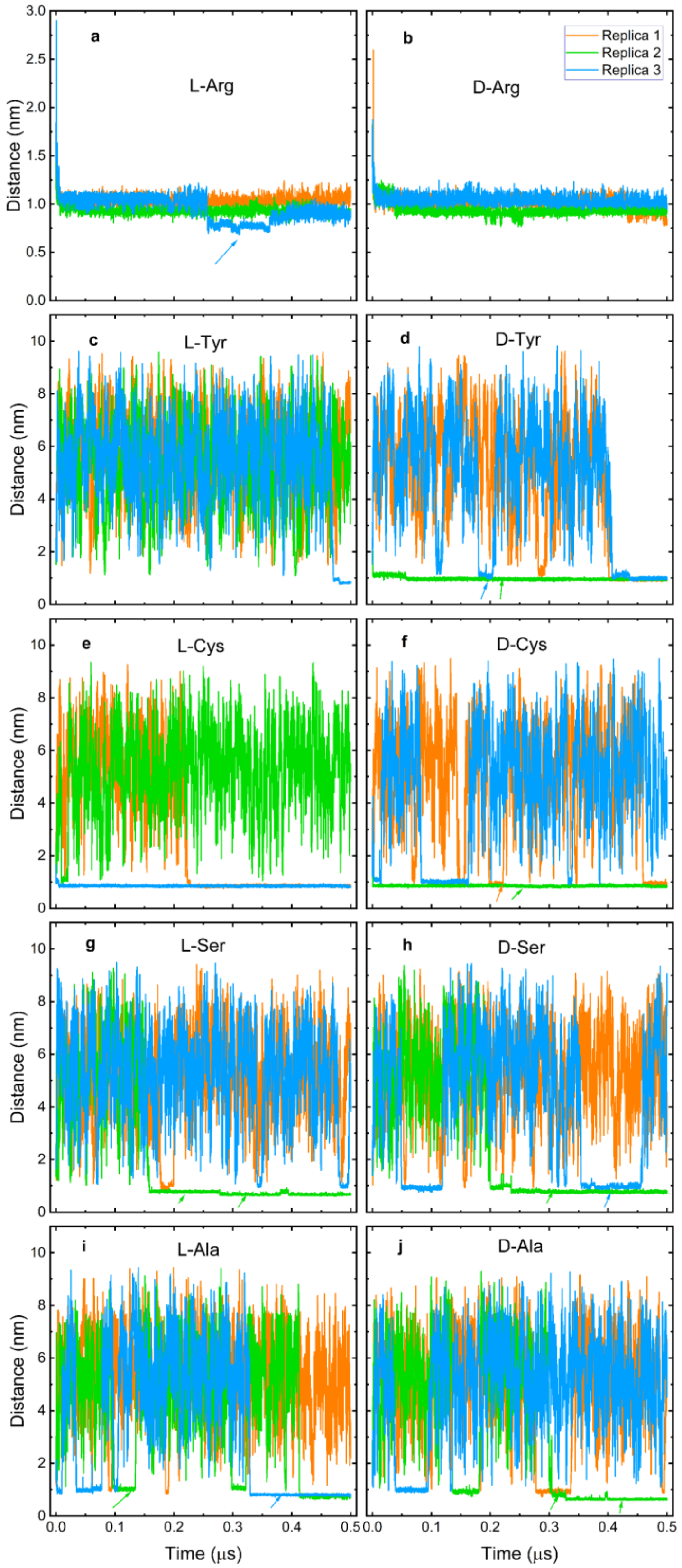

Fig. S4. The time evolution of COM distance between L-/D-amino acids and $Au_{38}(pMBA)_{24}$. The data was collected from three independent MD replicas.

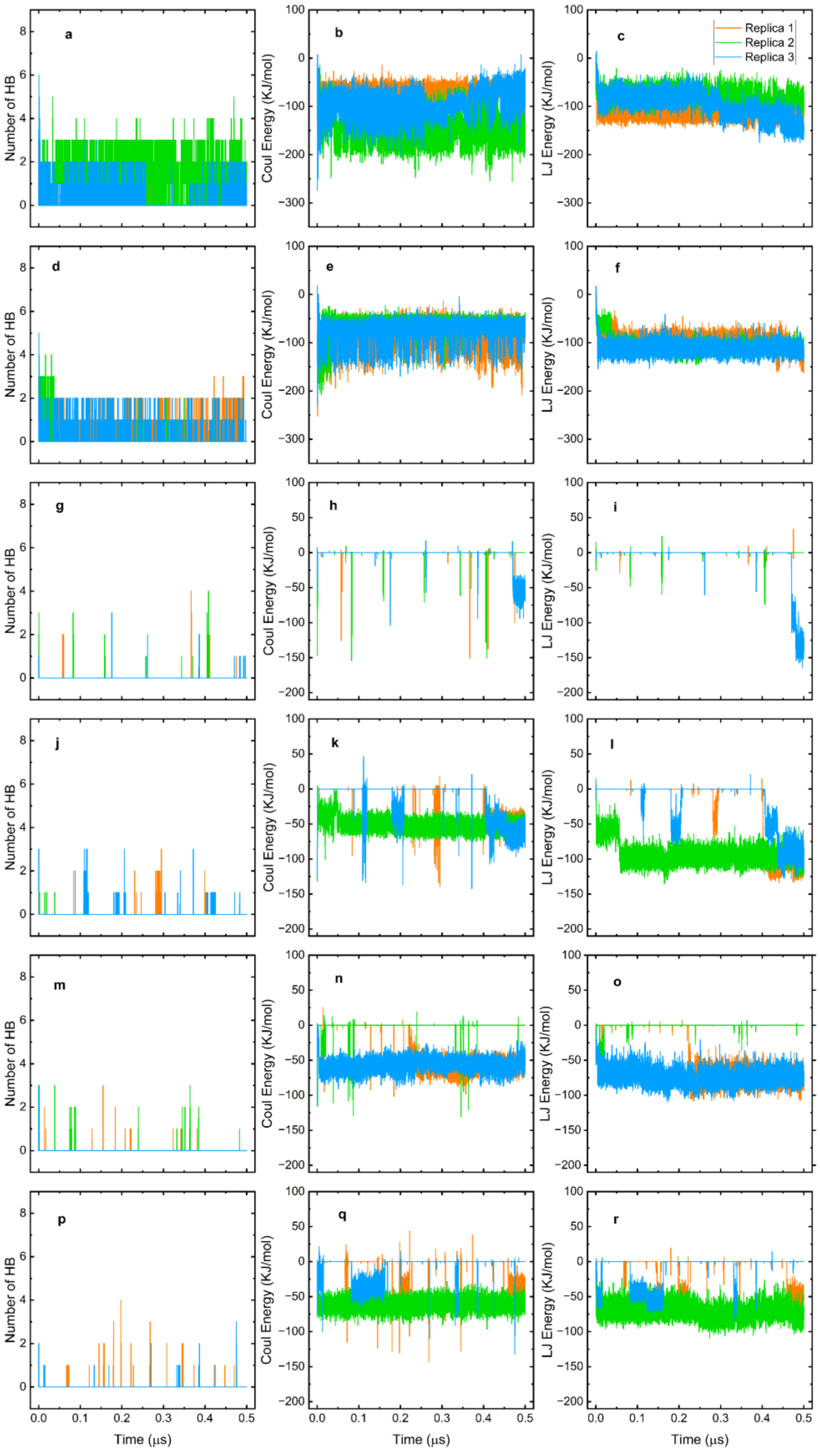

Fig. S5. 38*p*MBA nanoclusters and analytes. (a) The number of HB between L-Arg and $Au_{38}(pMBA)_{24}$, (b) the Coul and (c) LJ energy between L-Arg and $Au_{38}(pMBA)_{24}$, (d) the number of HB between D-Arg and $Au_{38}(pMBA)_{24}$, (e) the Coul and (f) LJ energy between D-Arg and $Au_{38}(pMBA)_{24}$, (g) the number of HB between L- Tyr and

$Au_{38}(pMBA)_{24}$, (h) the Coul and (i) LJ energy between L- Tyr and $Au_{38}(pMBA)_{24}$, (j) the number of HB between D-Tyr and $Au_{38}(pMBA)_{24}$, (k) the Coul and (l) LJ energy between D- Tyr and $Au_{38}(pMBA)_{24}$, (m) the number of HB between L- Cys and $Au_{38}(pMBA)_{24}$, (n) the Coul and (o) LJ energy between L- Cys and $Au_{38}(pMBA)_{24}$, (p) the number of HB between D- Cys and $Au_{38}(pMBA)_{24}$, (q) the Coul and (r) LJ energy between D- Cys and $Au_{38}(pMBA)_{24}$ in three independent MD replicas.

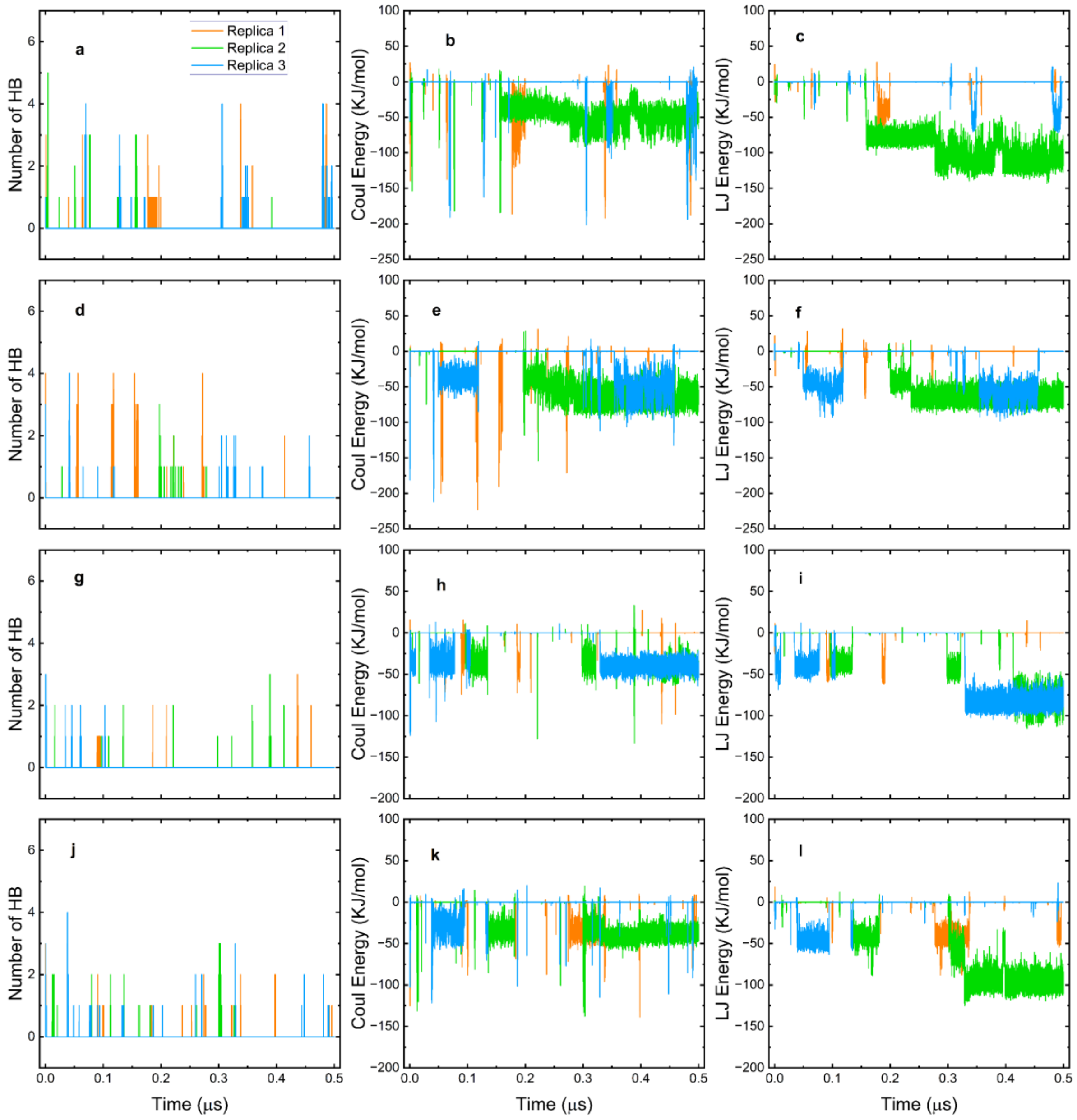

Fig. S6. 38*p*MBA nanoclusters and analytes. (a) The number of HB between L-Ser and $Au_{38}(pMBA)_{24}$, (b) the Coul and (c) LJ energy between L-Ser and $Au_{38}(pMBA)_{24}$, (d) the number of HB between D-Ser and $Au_{38}(pMBA)_{24}$, (e) the Coul and (f) LJ energy between D-Ser and $Au_{38}(pMBA)_{24}$, (g) the number of HB between L-Ala and $Au_{38}(pMBA)_{24}$, (h) the Coul and (i) LJ energy between L-Ala and $Au_{38}(pMBA)_{24}$, (j) the number of HB between D-Ala and $Au_{38}(pMBA)_{24}$, (k) the Coul and (l) LJ energy between D-Ala and $Au_{38}(pMBA)_{24}$ in three independent MD replicas.

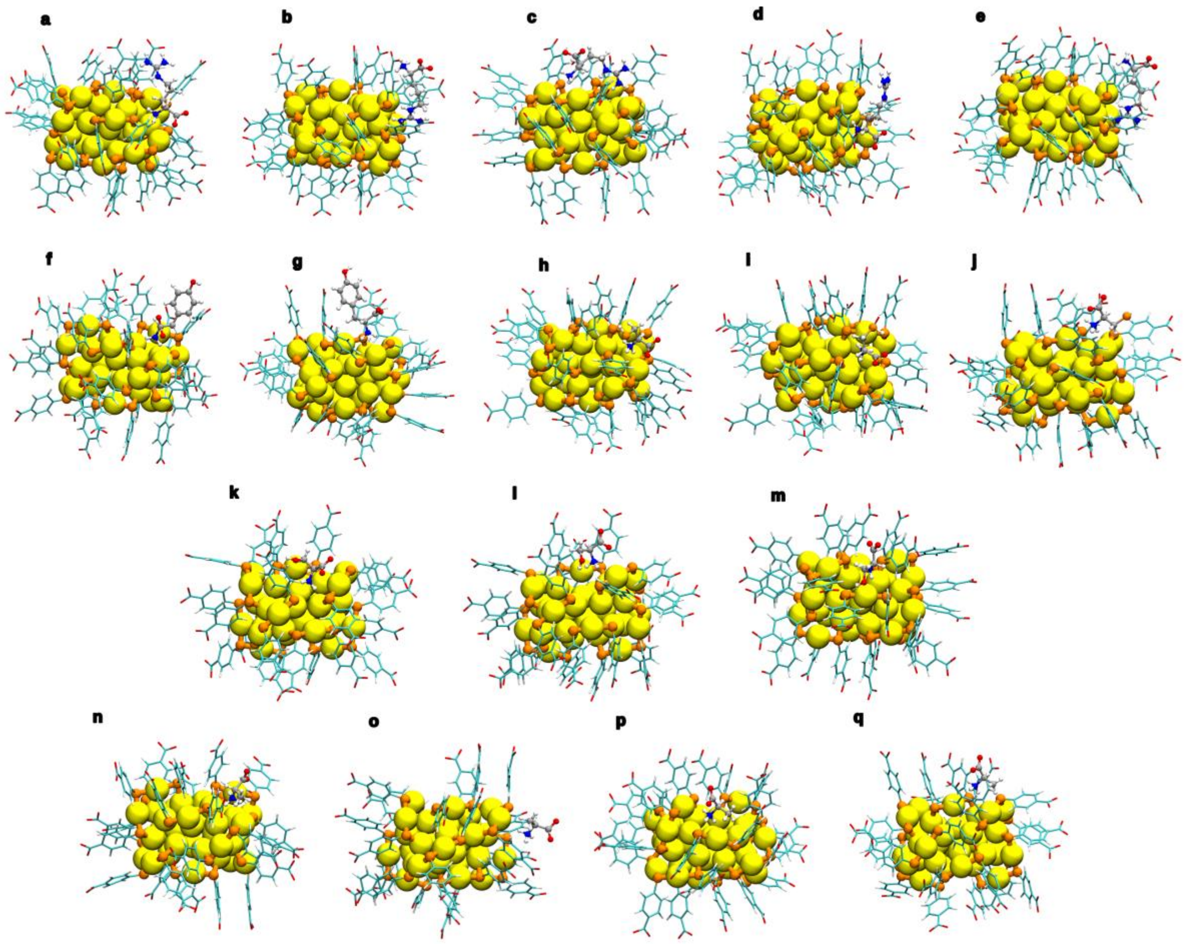

Fig. S7. Representative adsorbed L-/D- amino acids on $Au_{38}(pMBA)_{24}$. The representative structure of (a) L-Arg from geometric cluster (g-cluster) 1 at t=222.8 ns (second replica), (b) L-Arg from g-cluster 2 at t=339.7 ns (first replica), (c) L-Arg from g-cluster 5 at t=291.6 ns (third replica); and (d) D-Arg from g-cluster 1 at t=176.8 ns of second replica, (e) D-Arg from g-cluster 2 at 387 ns of third replica obtained by 1.25 Å RMSD geometric clustering. Representative structure of (f) D-Tyr from g-cluster 1 at t=325.7 ns of second replica, and (g) from g-cluster 4 at t=195.6 ns of the third replica; and (h) L-Cys from g-cluster 1 at t=426.5 ns from the third replica, and (i) D-Cys from g-cluster 1 at t=416.1 of the second replica, and (j) D-Cys from g-cluster 2 at t=202.3 ns of the first replica; and (k) L-Ser from g-cluster 1 at t=293 ns in the second replica, and (l) L-Ser from g-cluster 9 at t=178.4 ns of second replica, and (m) D-Ser from g-cluster 1 at t=306.8 ns of second replica, and (n) L-Ala from g-cluster 1 at t=352.4 ns in the third replica, and (o) the snapshot of L-Ala at t=134.1 ns in the second replica, and (p) D-Ala from g-cluster 1 at t=423.8 ns in the second replica, and (q) D-Ala from g-cluster 2 at t=331.2 ns in the second replica obtained by 1.5 Å RMSD geometric clustering of C_α of the amino acid with heavy atoms of $Au_{38}(pMBA)_{24}$. Colour code: Au=yellow; S=orange; O=red; N=blue; H=white; C (ligand layer)=cyan; C (analyte)=silver.

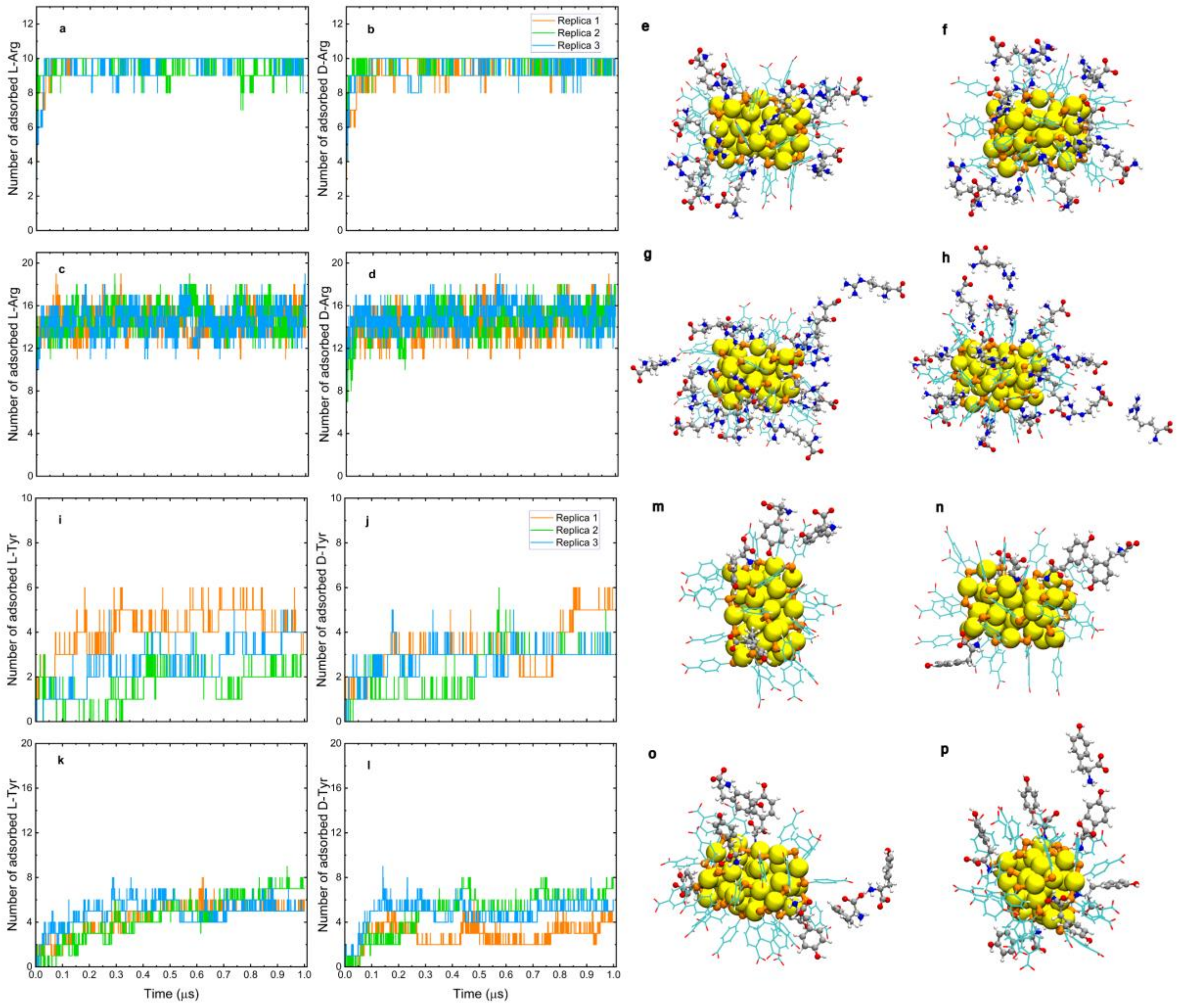

Fig. S8. Adsorbed L-/D-analytes on $Au_{38}(pMBA)_{24}$. Number of adsorbed analytes on $Au_{38}(pMBA)_{24}$ in the system of (a) 10 L-Arg, (b) 10 D-Arg, (c) 20 L-Arg and (d) 20 D-Arg molecules in three replicas during 1000 ns MD simulation. Screenshot from $Au_{38}(pMBA)_{24}$ with (e) 10 L-Arg molecules in the second replica at t=713 ns, (f) 10 D-Arg molecules in the third replica at t=771 ns, (g) 20 L-Arg molecules in the first replica at t=692 ns, and (h) 20 D-Arg molecules in the second replica at t=310 ns. Number of adsorbed analytes on $Au_{38}(pMBA)_{24}$ in the system of (i) 10 L-Tyr, (j) 10 D-Tyr, (k) 20 L-Tyr and (l) 20 D-Tyr molecules. $Au_{38}(pMBA)_{24}$ with (m) 10 L-Tyr molecules in the third replica at t=463 ns, (n) 10 D-Tyr molecules in the first replica at t=468 ns, (o) 20 L-Tyr molecules in the first replica at t=622 ns, and (p) 20 D-Tyr molecules in the second replica at t=891 ns. Colour code: Au=yellow; S=orange; O=red; N=blue; H=white; C (ligand layer)=cyan; C (analyte)=silver.

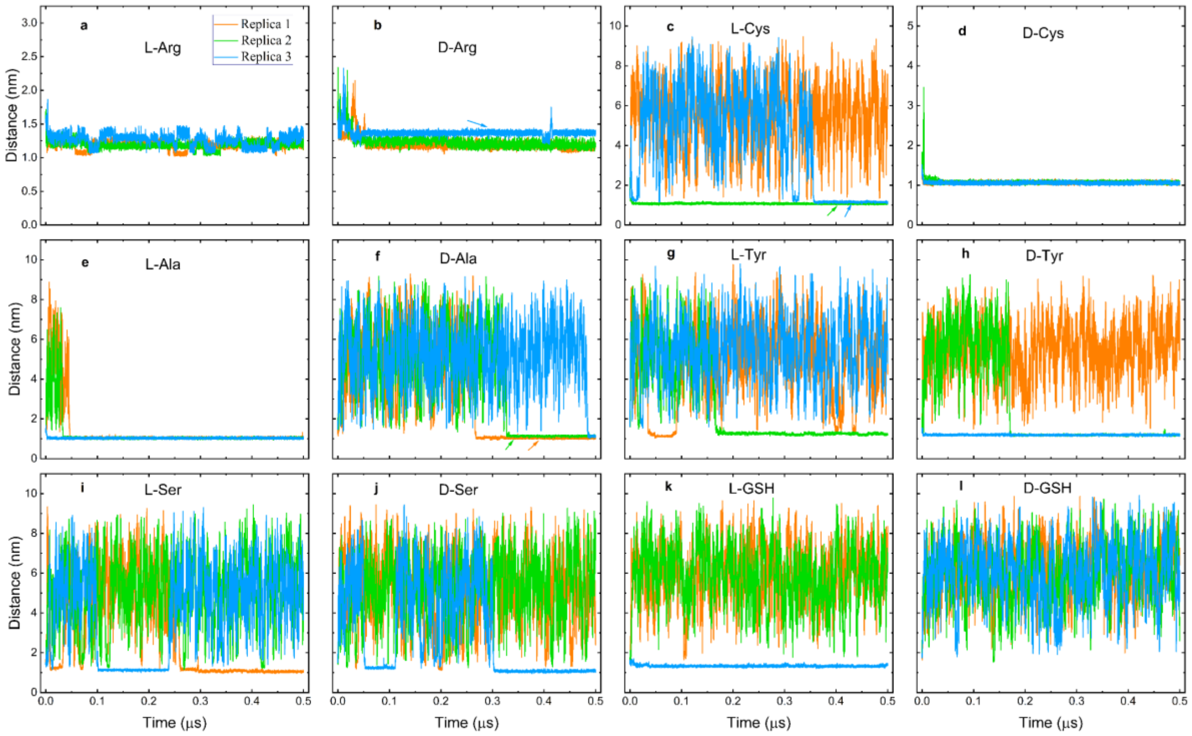

Fig. S9. The time evolution of COM distance between L-/D-amino acids and $Au_{102}(pMBA)_{44}$. The data was collected from three independent MD replicas.

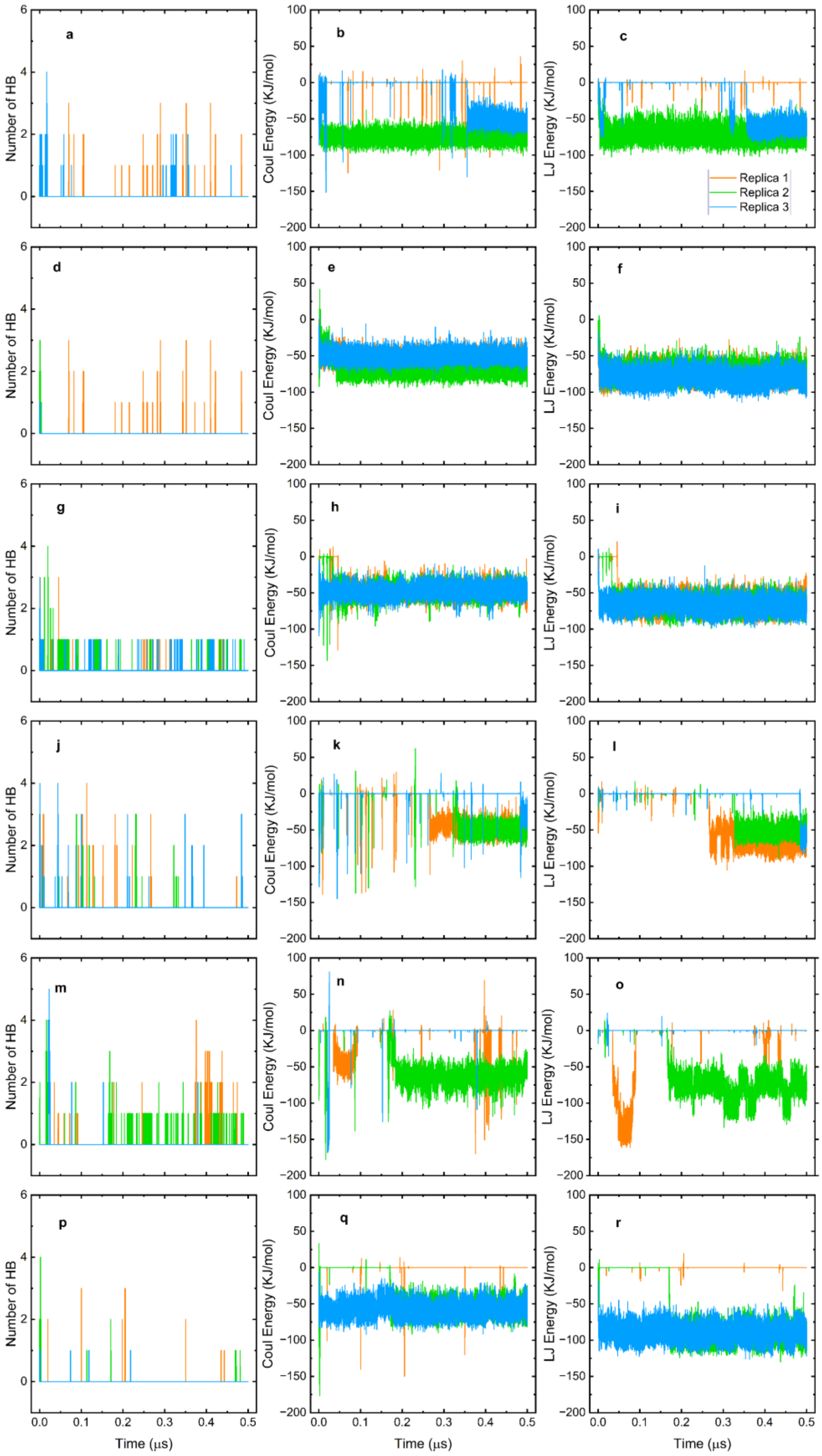

Fig. S10. 102*p*MBA nanoclusters and analytes. (a) The number of HB between L-Lys and $Au_{102}(pMBA)_{44}$, (b) the Coul and (c) LJ energy between L-Cys and $Au_{102}(pMBA)_{44}$, (d) the number of HB between D-Cys and $Au_{102}(pMBA)_{44}$, (e) the Coul and (f) LJ energy between D-Cys and $Au_{102}(pMBA)_{44}$, (g) the number of HB between

L-Ala and $Au_{102}(pMBA)_{44}$, (h) the Coul and (i) LJ energy between L-Ala and $Au_{102}(pMBA)_{44}$, (j) the number of HB between D-Ala and $Au_{102}(pMBA)_{44}$, (k) the Coul and (l) LJ energy between D-Ala and $Au_{102}(pMBA)_{44}$, (m) the number of HB between L-Tyr and $Au_{102}(pMBA)_{44}$, (n) the Coul and (o) LJ energy between L-Tyr and $Au_{102}(pMBA)_{44}$, (p) the number of HB between D-Tyr and $Au_{102}(pMBA)_{44}$, (q) the Coul and (r) LJ energy between D-Tyr and $Au_{102}(pMBA)_{44}$ in three independent MD replicas.

.

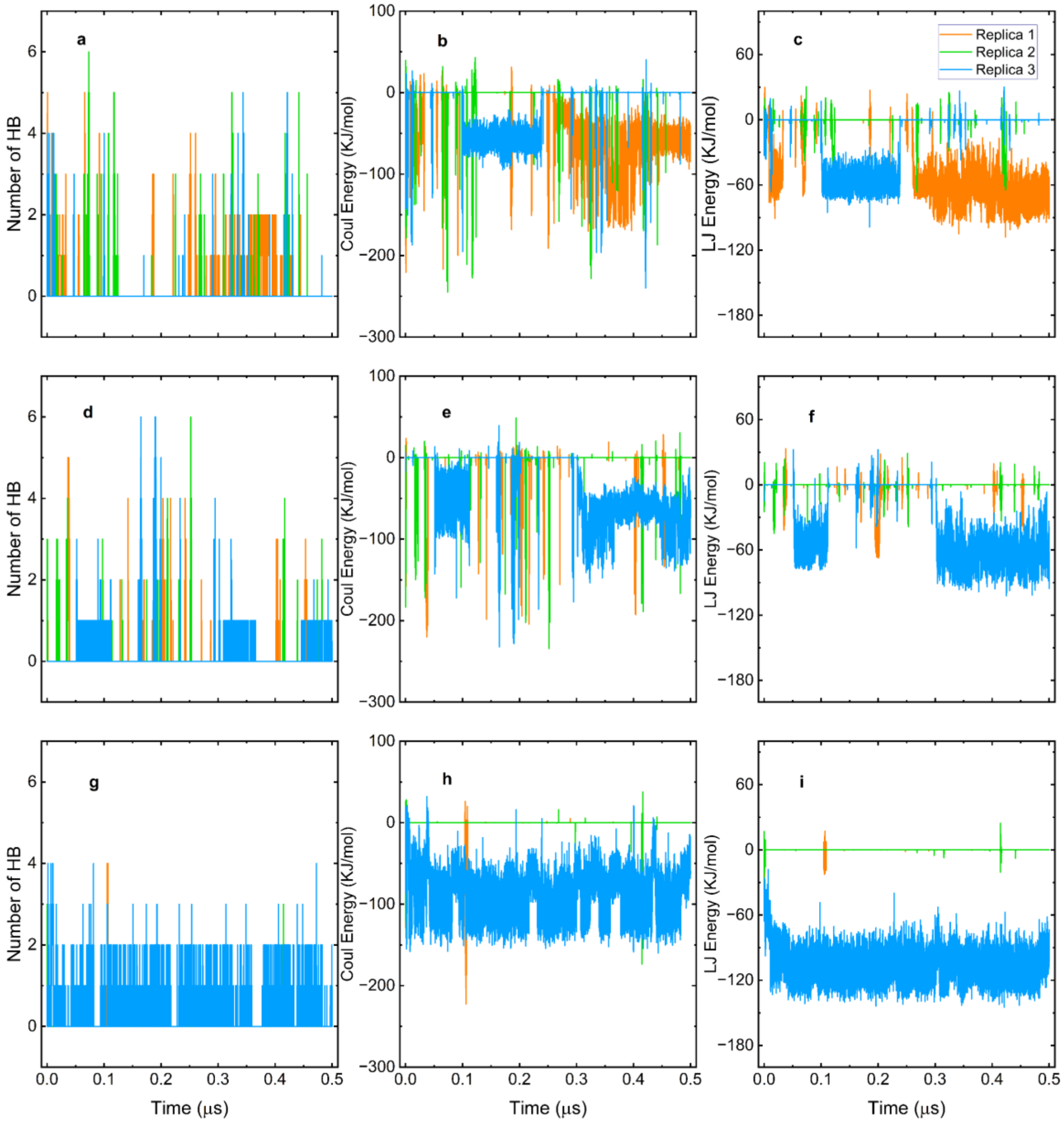

Fig. S11. 102*p*MBA nanoclusters and analytes. (a) The number of HB between L-Ser and $Au_{102}(pMBA)_{44}$, (b) the Coul and (c) LJ energy between L-Ser and $Au_{102}(pMBA)_{44}$, (d) the number of HB between D-Ser and $Au_{102}(pMBA)_{44}$, (e) the Coul and (f) LJ energy between D-Ser and $Au_{102}(pMBA)_{44}$, (g) the number of HB between L-GSH and $Au_{102}(pMBA)_{44}$, (h) the Coul and (i) LJ energy between L-GSH and $Au_{102}(pMBA)_{44}$ in three independent MD replicas.

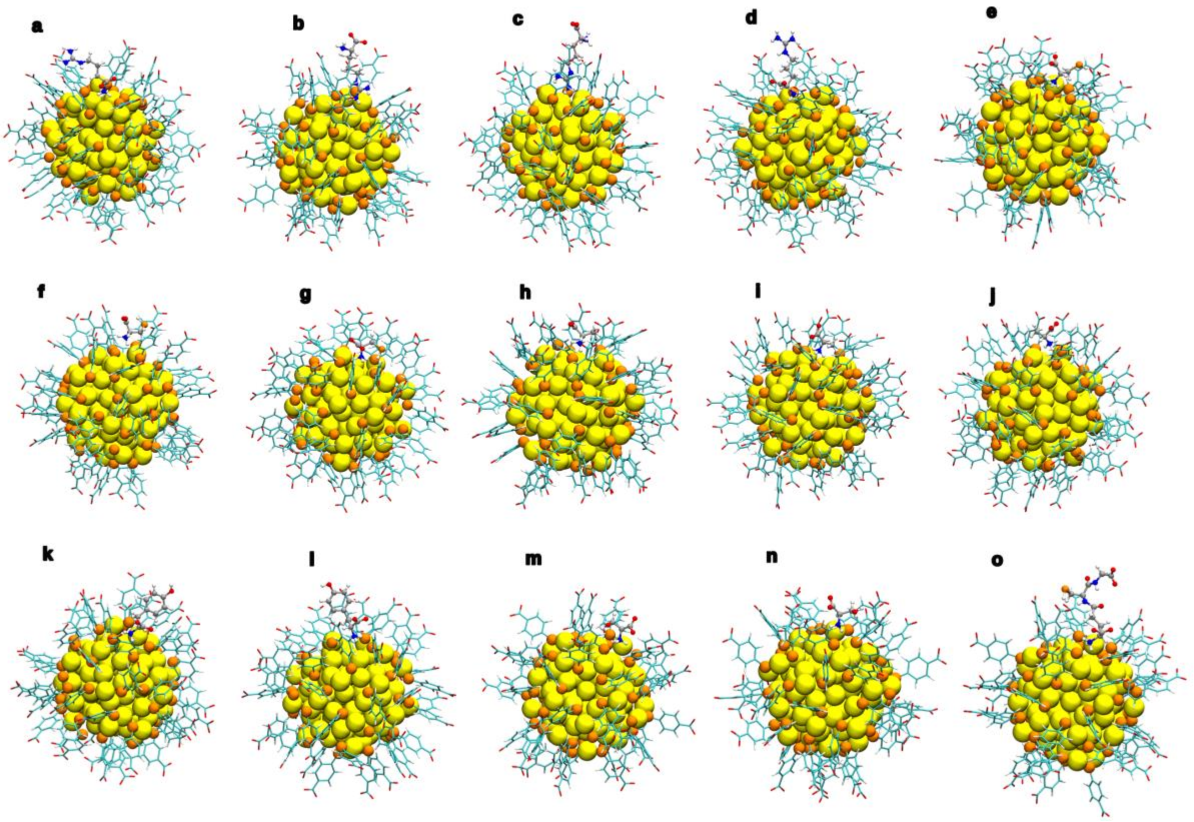

Fig. S12. **Representative adsorbed L-/D- amino acids on $Au_{102}(pMBA)_{44}$.** The representative structure of (a) L-Arg from g-cluster 1 at t=201.5 ns of first replica, (b) L-Arg from g-cluster 2 at t=191.4 ns of third replica; and (c) D-Arg from g-cluster 1 at t=456.6 ns of third replica, (d) D-Arg from g-cluster 2 at t=385.8 ns of first replica obtained by 1 Å RMSD geometric clustering of guanidine carbon and heavy atoms of AuNC. Representative structure of (e) L-Cys from g-cluster 1 at t=412.4 ns of replica 2, and (f) L-Cys from g-cluster 2 at t=403 ns of replica 3; and (g) D-Cys from g-cluster 1 at t=191.1 ns of the third replica; and (h) L-Ala from g-cluster 1 at t=161.4 ns in the second replica, and (i) D-Ala from g-cluster 1 at t=391 ns of first replica ,and (j) D-Ala from g-cluster 2 at t=338.3 ns of the second replica; and (k) L-Tyr from g-cluster 1 at t=287.7 ns of the second replica, and (l) D-Tyr from g-cluster 1 at t=245.2 ns of the third replica; and (m) L-Ser from g-cluster 1 at t=335.7 ns of first replica, and (n) D-Ser from g-cluster 1 at t=304.6 ns of third replica obtained by 1 Å RMSD geometric clustering of C_α of the amino acid with heavy atoms of $Au_{102}(pMBA)_{44}$, and (o) representative structure of L-GSH from g-cluster 1 at t=284 ns of third replica which has been obtained by 0.9 Å RMSD geometric clustering of 3 C_α of GSH with heavy atoms of $Au_{102}(pMBA)_{44}$ extracted from the third MD simulation replica. Colour code: Au=yellow; S=orange; O=red; N=blue; H=white; C (ligand layer)=cyan; C (analyte)=silver.

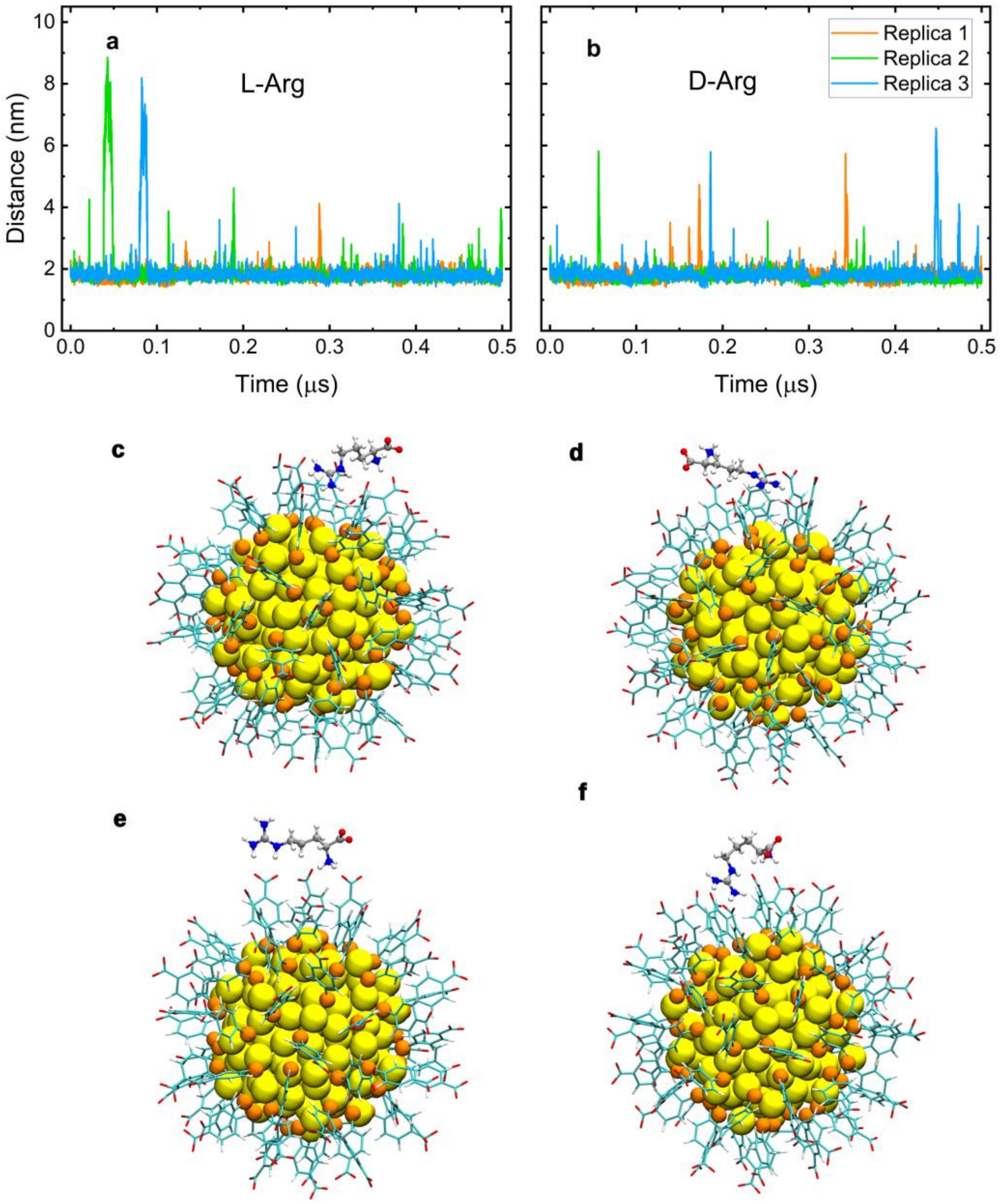

Fig. S13. The time evolution of COM distance between L-/D-Arg and $Au_{144}(pMBA)_{60}$, and representative adsorbed L-/D-Arg on $Au_{144}(pMBA)_{60}$. The distance between COM of (a) L- and (b) D-Arg with COM of $Au_{144}(pMBA)_{60}$ in three independent MD replicas. The representative structure of (c) L-Arg from g-cluster 1 at t= 405.6 ns of first replica, (d) L-Arg from g-cluster 2 at t=239 ns of second replica, (e) D-Arg from g-cluster 1 at t=225 ns of the first replica, and (f) D-Arg from g-cluster 2 at t=133.8 of the second replica obtained by 0.6 Å RMSD geometric clustering of guanidine carbon and heavy atoms of $Au_{144}(pMBA)_{60}$. Colour code: Au=yellow; S=orange; O=red; N=blue; H=white; C (ligand layer)=cyan; C (analyte)=silver.

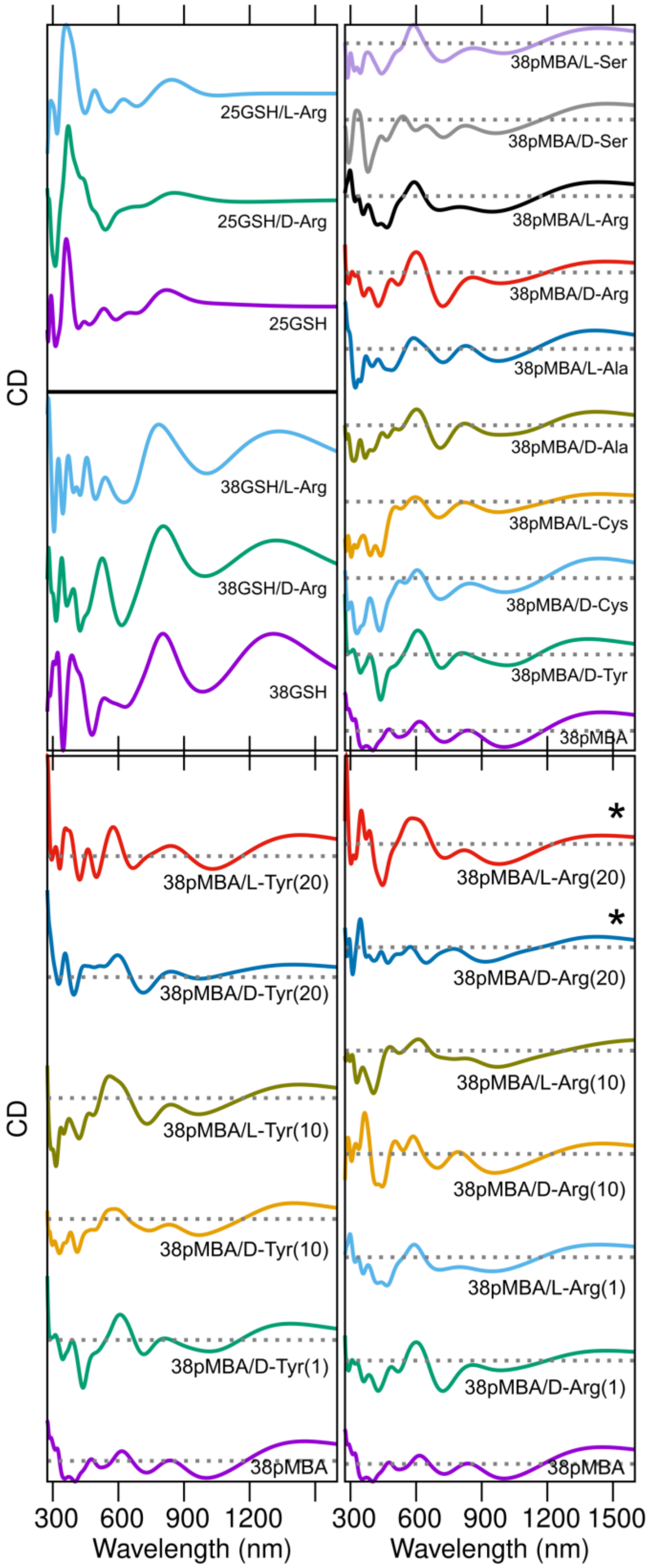

Fig. S14. Calculated CD spectra of 25GSH, 38GSH, and 38*p*MBA nanoclusters with chiral analytes. 38*p*MBA/L-Arg(20) and 38*p*MBA/D-Arg(20) have a reduced number of snapshots in statistical sampling due to computational limitations.

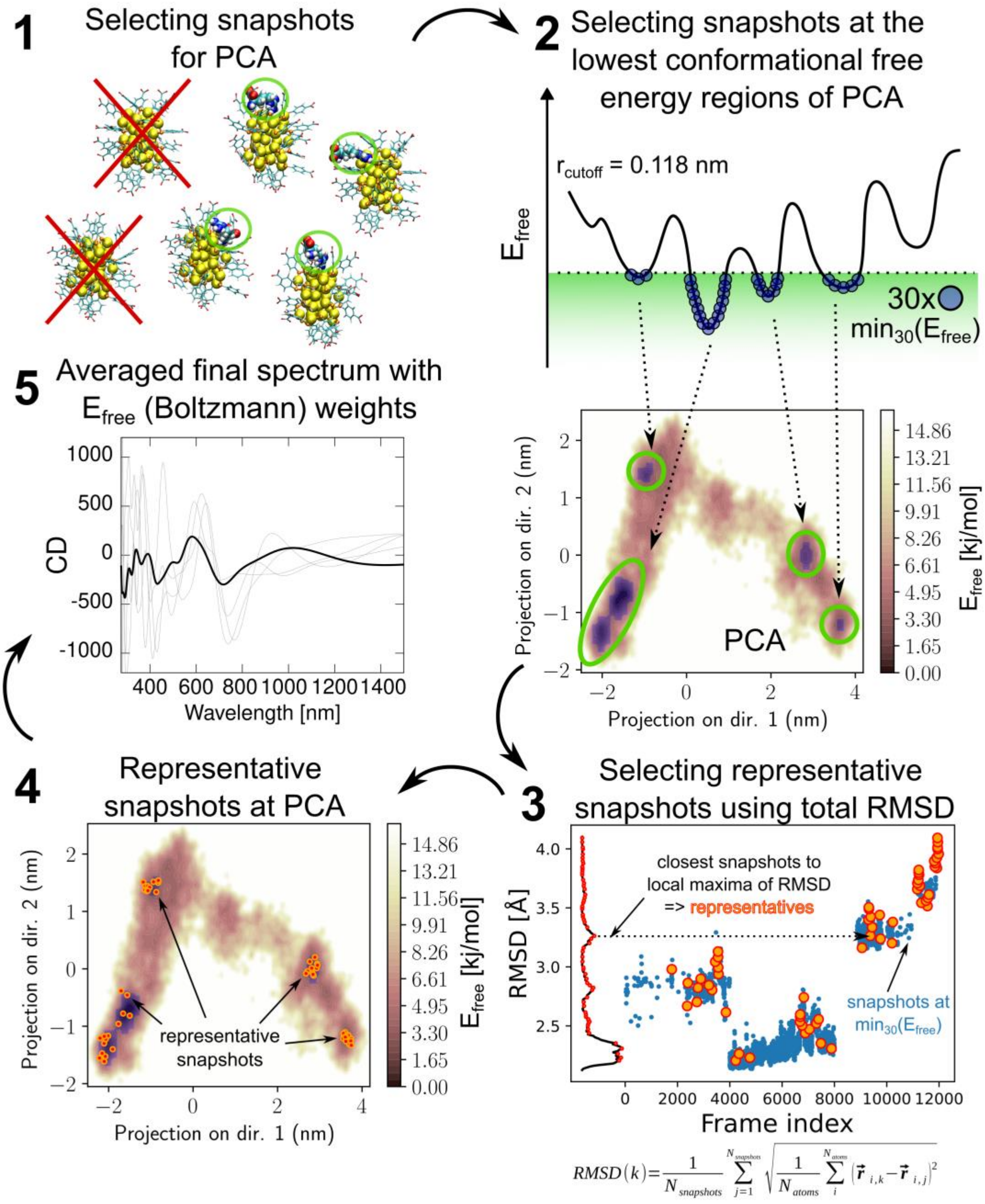

Fig. S15. Steps of the selection process of representative snapshots for statistical sampling of CD spectra. 1) selecting snapshots for PCA being either 1/5 evenly distributed from all MD-frames or then subset of snapshots showing interacting cluster-analyte hybrids (see Table S1), 2) calculation of PCA and conformational free energies (E_{free}) with respect to the two main PCA-eigenvectors and selection of snapshots under the lowest conformational free energy regions shaded in blue (=$min_{30}(E_{free})$). The 2D-space of PCA is divided into 100x100 bins and free energies are folded for the bins using Gaussian distribution with 0.05 nm broadening parameter at 300K. The free energies lower than 16.0 kJ/mol are examined and plotted with contours when fixing the minimum to 0 kJ/mol. The lowest free energy phase space is spanned using 30 cutoff circles of 0.118 nm radius, 3) calculation of total RMSD (see the equation in figure) distribution using Gaussians of 0.005 Å broadening parameter and selecting the representative snapshots from the closest MD-frames by position to the local maxima of RMSD distribution. RMSD analysis includes only the snapshots under the lowest E_{free} regions (blue shaded areas in step 2), 4) collecting conformational free energies and Boltzmann factors of the selected representative snapshots, and 5) calculating a weight averaged, statistically sampled CD spectra representing the final prediction. Examples of PCA and RMSD plots shown here are for 38pMBA/D-Arg.

Table S1: Numerical details of statistical sampling at different steps used to select representatives for the CD spectra. 38*p*MBA/L-Arg(20) and 38*p*MBA/D-Arg(20) systems have restricted number calculated representative snapshots in sampling of CD due to computational limitations.

System	Total number of snapshots in three replicas	Snapshots in PCA	Min. number of interacting analytes in PCA snapshots	Snapshots at the lowest E_{free} areas (percentages of snapshots in PCA)	Representative snapshots	Number of analytes in CD calc.
25GSH	60000	12000	-	5540 (46.2%)	30	0
25GSH/D-Arg	60000	12000	-	3679 (30.7%)	32	0-1
25GSH/L-Arg	60000	12000	-	3836 (32.0%)	29	0-1
38GSH	60000	12000	-	4946 (41.2%)	21	0
38GSH/D-Arg	60000	12000	-	5342 (44.5%)	15	0-1
38GSH/L-Arg	60000	12000	-	3143 (26.2%)	18	0-1
38*p*MBA	60000	12000	-	5032 (41.9%)	36	0
38*p*MBA/D-Tyr	60000	10000	1	4391 (43.9%)	34	1
38*p*MBA/D-Cys	60000	13149	1	4374 (43.7%)	29	1
38*p*MBA/L-Cys	60000	11282	1	3247 (28.8%)	21	1
38*p*MBA/D-Ala	60000	12517	1	5190 (41.5%)	26	1
38*p*MBA/L-Ala	60000	14333	1	6510 (45.4%)	24	1
38*p*MBA/D-Arg	60000	12000	1	4368 (36.4%)	60	1
38*p*MBA/L-Arg	60000	12000	1	4527 (37.7%)	29	1
38*p*MBA/D-Ser	60000	10477	1	3832 (36.6%)	34	1
38*p*MBA/L-Ser	60000	9513	1	2888 (30.4%)	23	1
38*p*MBA/D-Arg(10)	135000	22500	7	9993 (44.4%)	14	9-10
38*p*MBA/L-Arg(10)	135000	22500	7	11944 (53.1%)	31	9-10
38*p*MBA/D-Arg(20)	135000	22418	12	8777 (39.2%)	7 (out of 21)	14-16
38*p*MBA/L-Arg(20)	135000	22449	12	12352 (55.0%)	7 (out of 21)	15-17
38*p*MBA/D-Tyr(10)	135000	28573	3	12293 (43.0%)	22	3-4
38*p*MBA/L-Tyr(10)	135000	24747	3	6501 (26.3%)	19	3-5
38*p*MBA/D-Tyr(20)	135000	22649	5	8046 (35.5%)	40	5-8
38*p*MBA/L-Tyr(20)	135000	20331	5	10280 (50.6%)	17	5-8
